# To kill or to be killed: pangenome analysis of *Escherichia coli* strains reveals a tailocin specific for pandemic ST131

**DOI:** 10.1186/s12915-022-01347-7

**Published:** 2022-06-16

**Authors:** Erwin Tantoso, Birgit Eisenhaber, Miles Kirsch, Vladimir Shitov, Zhiya Zhao, Frank Eisenhaber

**Affiliations:** 1 Genome Institute of Singapore (GIS), Agency for Science, Technology and Research (A*STAR), 60 Biopolis Street, Singapore, 138672 Republic of Singapore; 2 Bioinformatics Institute (BII), Agency for Science, Technology and Research (A*STAR), 30 Biopolis Street #07-01, Matrix Building, Singapore, 138671 Republic of Singapore; 3 Present address: Northeastern University, Boston, USA; 4 Present address: The University of Cambridge, Cambridge, UK; 5 School of Biological Sciences (SBS), Nanyang Technological University (NTU), 60 Nanyang Drive, 637551 Singapore, Republic of Singapore

**Keywords:** *Escherichia coli*, Pangenome, Softcore genome, ST11, ST131 pathogenic strain, Prophage, R2-pyocin, Tailocin, CoinFinder, Pandemic *E. coli*

## Abstract

**Background:**

*Escherichia coli* (*E. coli*) has been one of the most studied model organisms in the history of life sciences. Initially thought just to be commensal bacteria, *E. coli* has shown wide phenotypic diversity including pathogenic isolates with great relevance to public health. Though pangenome analysis has been attempted several times, there is no systematic functional characterization of the *E. coli* subgroups according to the gene profile.

**Results:**

Systematically scanning for optimal parametrization, we have built the *E. coli* pangenome from 1324 complete genomes. The pangenome size is estimated to be ~25,000 gene families (GFs). Whereas the core genome diminishes as more genomes are added, the softcore genome (≥95% of strains) is stable with ~3000 GFs regardless of the total number of genomes. Apparently, the softcore genome (with a 92% or 95% generation threshold) can define the genome of a bacterial species listing the critically relevant, evolutionarily most conserved or important classes of GFs. Unsupervised clustering of common *E. coli* sequence types using the presence/absence GF matrix reveals distinct characteristics of *E. coli* phylogroups B1, B2, and E. We highlight the bi-lineage nature of B1, the variation of the secretion and of the iron acquisition systems in ST11 (E), and the incorporation of a highly conserved prophage into the genome of ST131 (B2). The tail structure of the prophage is evolutionarily related to R2-pyocin (a tailocin) from *Pseudomonas aeruginosa* PAO1. We hypothesize that this molecular machinery is highly likely to play an important role in protecting its own colonies; thus, contributing towards the rapid rise of pandemic *E. coli* ST131.

**Conclusions:**

This study has explored the optimized pangenome development in *E. coli*. We provide complete GF lists and the pangenome matrix as supplementary data for further studies. We identified biological characteristics of different *E. coli* subtypes, specifically for phylogroups B1, B2, and E. We found an operon-like genome region coding for a tailocin specific for ST131 strains. The latter is a potential killer weapon providing pandemic *E. coli* ST131 with an advantage in inter-bacterial competition and, suggestively, explains their dominance as human pathogen among *E. coli* strains.

**Supplementary Information:**

The online version contains supplementary material available at 10.1186/s12915-022-01347-7.

## Background

*Escherichia coli* is one of the most well-known commensal Gram-negative bacteria, which is commonly associated with the gut microbiome. Since first identified in 1844, it has been widely studied as a model organism in the laboratory. However, recent findings have shown not only the versatility of *E. coli* living in different ecological niches but also the diversity of its genotypes including strains with pathogenicity for animals and human [1, 2]. *Escherichia coli* has been implicated in several disease outbreaks involving food contamination and diarrhea [3–7]. It is also one of the bacteria most commonly isolated from the urine of patients suffering from urinary tract infection (UTI) worldwide [8–11]. Recently, the ST131 strains, some of the most prominent variants of *E. coli*, have risen quickly to become pandemic with a multidrug-resistant phenotype [12, 13]. All these evidences suggest that *E. coli* is not simply a model organism but it has implications for public health [14, 15], which need attention with regard to the mechanisms of pathogenicity [16, 17].

*Escherichia coli* is known to inhabit the lower intestinal tract of warm-blooded animals, including human. It can be discharged through fecal material to the living environment, particularly soil and water, which could have public health implications [15, 18]. This environmental *E. coli* can then adapt to new environmental habitats by acquiring genes, virulence factors, and mobile genetic elements through horizontal gene transfer with the environmental bacteria [19].

There is no obvious association of *E. coli* phylogroups with the geographical location, as well as the living areas and feeding habits [20]. Nonetheless, existing studies have shown that the phylogroups A and B1 can be isolated from multiple hosts as well as environment [21–26]. Phylogroups B2 and D are usually extraintestinal pathotypes [27–29]. Phylogroup E, particularly O157, is usually isolated from contaminated food [30]. To date, the different pathotypes of *E. coli* can be isolated from multiple hosts and the commonly associated phylogroups are summarized by Denamur et al. [31].

Depending on the location or site where pathogenic *E. coli* is isolated, it can be broadly categorized into intestinal pathogenic *E. coli* (InPEC) [32] or extraintestinal pathogenic *E. coli* (ExPEC) [33]. The pathogenic *E. coli* strains can be further classified by pathotypes. InPEC is categorized into several major groups, namely AIEC (Adherent Invasive *E. coli*), EHEC (Enterohemorrhagic *E. coli*), EAEC (Enteroaggregative *E. coli*), ETEC (Enterotoxigenic *E. coli*), EPEC (Enteropathogenic *E. coli*), and DAEC (Diffusely Adherent *E. coli*). On the other hand, several notable ExPEC are UPEC (Urinary Pathogenic *E. coli*), NMEC (Neonatal Meningitis-associated *E. coli*), and APEC (Avian Pathogenic *E. coli*). Different pathotypes have their own associated virulence factors and disease manifestations that have been summarized in several publications [32, 34, 35]. Virulence factor typing was attempted to be used for predicting the pathotypes of *E. coli*. However, there are often ambiguities [35–38] as some pathogenic *E. coli* share similar virulence factors. For example, InPECs share similar virulence factors with similar pathology within the same subgroup, whereas ExPECs frequently do not even have specific virulence factors that define a given subtype [35].

Rasko et al. [39] performed a first comparative analysis of 17 *E. coli* genomes available in 2008 and showed that the *E. coli* pathotypes can be distinguished by using a limited set of molecular markers that are annotated as pilus or fimbrial components as well as their secretion system. Clark et al. [34] constructed a pathotype database with 107 *E. coli* genomes showing presence/absence of selected virulence factors. They found a trend of certain pathotype-associated virulence factors correlating with evolutionarily related groups of *E. coli* strains (phylogroups). Notably, all these studies do not provide a comprehensive characterization of *E. coli* subtypes but instead just rely on known virulence factor lists for classification. Undoubtedly, the problem of virulence factors’ overlap across the different pathotypes remained unsolved in these analyses.

To date, the largest study of *E. coli* genomes (more than 10,000 including incomplete ones) has been reported by Horesh et al. [40]. They provide a classification of *E. coli* lineages according to sequence types (STs; defined by multi-locus sequence typing of seven housekeeping genes) and phylogroups. Horesh et al. noted that their collection is severely biased towards *E. coli* strains of clinical significance. In fact, the two largest lineages are the collections of pathogenic ST11 and ST131 *E. coli* strains, which belong to phylogroups E and B2, respectively. Therefore, efforts should be taken to sample a more diverse collection of *E. coli* genomes.

The available literature about pangenome analyses [41] of *E. coli* revealed several surprising insights: (i) The *E. coli* genomes are very diverse and less than 1000 genes of any specific *E. coli* genome are shared across the species (core genome) [42], while tens of thousands of genes are considered part of the accessory genome shared by only a limited number of strains. (ii) With the availability of an increasing number of *E. coli* genomes, we see the pangenome size increasing while the core genome size keeps decreasing. This can be seen from the published analyses of 17 genomes [39], 61 genomes [42], 186 genomes [43], and 307 genomes [44]. The pangenome size increased from ~13,000 to ~23,000 genes. At the same time, the core genome size fell from ~2200 to ~800 genes in these studies. Due to the diversity of *E. coli* living environments, it is expected that further genome sequencing will continue the trends [45]. To note, the core genome is generally expected to represent the essential genes of *E. coli* [46]. However, the definition of the core genome (as genes shared by all the genomes) is apparently too stringent and, therefore, several authors experimented with softcore genome definitions (the set of genes shared by a certain percentage of genomes) [43, 47].

The pangenome construction critically depends on identifying clusters of homologous genes/proteins or gene/protein families (GF) among all the genomes in the study [39, 42–44, 48]. Various publications have used different criteria of defining clusters of homologous genes; however, two most important parameters are the sequence identity (SeqID) and sequence length coverage (SeqLC) in the pairwise alignment of two protein sequences. Based on these thresholds, a binary decision (belonging or not belonging to a GF) is taken. It has been shown that too stringent criteria lead to overestimation of cluster numbers, while too relaxed criteria put unrelated genes/proteins into the same cluster and underestimate the pangenome size [48]. Whereas previously published studies have taken arbitrary, ad hoc thresholds, finding the optimal parameters (with a criterion such as the Jaccard similarity index for a comparison of two or more methods for sequence homology assignment) should be used for this purpose. An exhaustive search in the SeqID and SeqLC parameter space was published for the pangenome expansion of *Streptococcus pyogenes* [49] with an optimum for SeqID=50…60% and SeqLC=60%. Finally, the presence/absence matrix (PAM; with GFs and genomes as indices) with values of 1 (indicates presence of GF in the genome) and 0 (indicates absence of GF in the genome), respectively, can be determined from the gene/protein list in the GFs.

The pangenome matrix can be utilized to find relationships between *E. coli* genomes, particularly for creating a pangenome tree of *E. coli* [43]. More importantly, we expect that the pangenome matrix can be used for molecular characterization of different subtypes of *E. coli*. The frequency or distribution of a gene family across all the genomes is expected to follow U-shape distribution. As previous pangenome studies have shown [50], most gene families are either singletons or commonly shared across genomes. Thus, for the characterization of subgroups of *E. coli* strains, most gene families are not informative except for those in the accessory genome.

Putting all perspectives together, in this study, we aim at performing (1) the construction of the *E. coli* pangenome with a careful preprocessing of genomes and a systematic search for optimal pangenome parametrization; (2) the characterization of *E. coli* subtypes at the level of gene and biomolecular mechanism occurrences, particularly phylogroups, sequence types, and virulence factors; and (3) in-depth analysis of specific, insufficiently characterized gene families in the distinct *E. coli* subtypes for the discovery of their actual biological function. This analysis provides an unparalleled insight into distinctive molecular characteristics of various subtypes of *E. coli* that explain hitherto not understood biological differences between groups of these bacterial strains.

## Results

### Characteristics of the *E. coli* genomes

As described in the “Methods” section below, we extracted *E. coli* genome sequences and their annotations from public repositories. We applied a clean-up procedure to ensure data quality and to suppress redundancy. In the final set of 1622 *E. coli* genomes, the number of nucleic acid sequences per genome ranges from 1 to 14, with 389 genomes containing only chromosomal sequences with no plasmid sequence and 1233 genomes containing at least one plasmid sequence. The genomic sequence length ranges from 4,456,672 to 6,162,737 bp with an average GC content of 50.65%. The total number of protein sequences per genome ranges from 3973 to 5618 sequences. The number of proteins is highly correlated with the genomic length with correlation coefficient of 0.9776.

Given the genome sequences, we performed in silico sequence typing, phylotyping, and serotyping for each genome as described in the “Methods” section. We detected 18 genomes with unknown sequence type (in addition to 385 sequence types for all remaining genomes) and 176 genomes with ambiguous H-serotypes (H-unknown; all other genomes have defined O- and H-serotypes). There are eight major phylogroups of *E. coli* (A, B1, B2, C, D, E, F, and G [51, 52]) that cover all but four genomes that are outliers in the phylogenetic tree (one belongs to clade I, two are classified as E or clade I, and one genome is unknown). The distribution of genomes among the most important sequence types and phylogroups (with at least 10 genomes) has been illustrated in Additional file 1: Fig. S1.

Additional file 1: Figure S2 shows the phylogroups’ genome sizes as well as proteome sizes. As a trend, phylogroup A of *E. coli* has the smallest genome/proteome size whereas the phylogroup E has the largest. The phylogroup B1 has an especially wide range of genome sizes. This is probably due to the presence of two distinct groups in phylogroup B1 of *E. coli*, which will be discussed later.

Our results show that the pairwise average nucleotide identity (ANI) across the genomes is not below 95%, which indicates that all genomes are from the same species [53]. We used the pairwise ANI to exclude sequentially redundant genomes (with more than 99.99% similarity as described in the “Methods” section). This has led to the total number of genomes for further analysis to be 1324. The detailed information regarding retained and removed genomes is available in Supplementary file 1 (as part of zip package Additional file 3) together with the information about serotypes, sequence types, and phylogroups.

Additional file 1 Figure S3 displays the distribution of sequence types and phylogroups in the remaining 1324 genomes. Out of the 385 sequence types (ST) available, 364 of them are represented by fewer than 10 genomes. For 21 STs, we find at least 10 genomes. The top 3 sequence types are ST10, ST11, and ST131. The *E. coli* K-12 belongs to ST10, whereas ST11 includes the O157 EHEC strain and ST131 is one of the important subtypes manifesting multidrug resistance. Similarly to what has been reported previously, these three STs are the dominant ones in the genome collection [40]. Focusing on STs with at least 10 genomes, these 21 STs include a total of 674 genomes. This represents 50.91% of the total number of genomes. In terms of phylogroups’ prevalence, the genomes are dominated by phylogroups A, B1, and B2 followed by phylogroups E, D, F, C, and G.

### The different subtypes of *E. coli* and their relevance to virulence

Several studies have distinguished the different pathotypes or subsets of *E. coli* strains according to virulence factors [34, 39, 54]. However, the lists of virulence factors are expected to be incomplete. Though some phylogroups are more associated with certain pathotypes, we find that certain virulence factors are not as specific [29] as previously described. For example, the colonization factor antigen I (CFA/I), which is often associated with ETEC strains, is also present in some of the EHEC, APEC, and nonpathogenic strains [20]. Further, the Nissle 1917 strain known to be a commensal one shares many virulence factors with ExPEC strains (though it does not have some other of the ExPEC virulence factors such as *hlyA* and *pap*).

Taken together, we think that the prediction of potential pathogenicity of *E. coli* is more relevant than the exact pathotype assignment. Therefore, we classified the potential pathogenicity (likelihood of virulence) of *E. coli* genome according to its virulence factor count instead. Based on the number of virulence factors present in the *E. coli* genome, the virulence category is defined as likely nonpathogenic, likely virulent, highly virulent, and extremely virulent (see “Methods”).

**Fig. 1:**
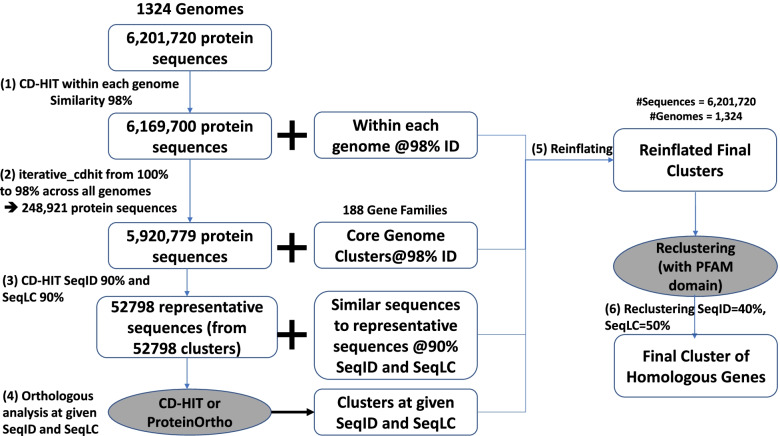
Steps in pangenome development. The complete genomes of *E. coli* (1324 genomes) with a total of 6,201,720 protein sequences are evaluated through multiple steps, i.e., (1) within-genome cluster analysis to extract representative (longest) protein sequences with at least 98% identity; (2) across-genome analysis to extract core genome sequences with at least 98% identity; (3) across-genome analysis to extract representative sequences from clusters of homologous sequences at SeqID=90% and SeqLC=90%; (4) parametrization of representative sequences from step (3) across different SeqID [range 40 to 80%] and SeqLC [range 50 to 90%] to obtain clusters of homologous genes/proteins; (5) put all the clusters together from step (1) to (4); and (6) merging of clusters with identical PFAM domains through re-clustering of representative sequences from each cluster from step (5) with SeqID=40% and SeqLC=50%

Figure 1 shows the distribution of virulence categories in the different phylogroups. It is clear that each phylogroup has genomes with different levels of virulence category. Phylogroups B2 (including both non-ST131 and ST131 strains), E, and G are overrepresented with genomes of higher-level virulence categories. Thus, these three phylogroups are the most likely ones to have pathogenic strains. The phylogroups D and F have a moderately high number of virulence genes. On the other hand, the phylogroups A, B1, and C have overwhelmingly low virulence strains. This is in concordance with existing knowledge that phylogroups A and B1 tend to belong to commensal strains of *E. coli* [55, 56]. The phylogroup C (though commonly associated with APEC strains, avian pathogens) is phylogenetically close to phylogroups A and B1 [31].

Further, we focus on the virulence analysis among the more common sequence types in *E. coli* genome, which have at least 10 genomes. Additional file 1: Figure S4 shows the distribution of virulence categories in the different sequence types. It can be seen that there are cases of quite different levels of virulent strains within the same phylogroup. It is interesting to note that the *E. coli* ST131 strains generally have fewer virulence factors compared to some other sequence types in the same phylogroup B2; thus, the virulence of ST131 strains is apparently not primarily driven by the number of known virulence factors.

### Optimized parameters for finding clusters of homologous genes

**Fig. 2:**
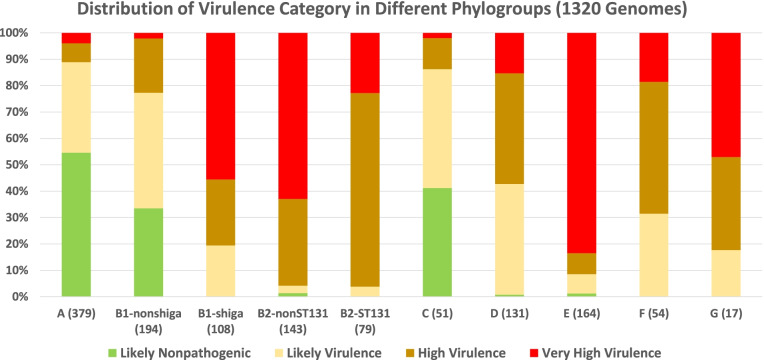
The distribution of virulence categories across the eight phylogroups of *E. coli*. Based on the total number of virulence factors (VFs) present in the genome, we categorized the genome into four virulence categories, i.e., (1) likely nonpathogenic (#VFs <6); (2) likely virulent (6 ≤ #VFs <14); (3) highly virulent (14 ≤ #VFs < 22), and (4) extremely virulent (#VFs ≥ 22)

Despite the pangenome profiling of *E. coli* has previously been attempted several times [39, 42–44, 48], there is no standardized protocol with optimized parameters for identifying clusters of homologous genes or GFs. We followed the sequential steps of a previously validated protocol [49] for pangenome development. Details for the *E. coli* data set are shown in Fig. 2 and are described in the “Methods” section below. We exhaustively scanned the parameter space for SeqID and SeqLC for homologous clustering of protein sequences. Additional file 2: Table S1 shows the total number of clusters identified with CD-HIT and ProteinOrtho as well as the Jaccard similarity indices across different ranges of SeqID and SeqLC. While it is reasonable to expect a higher Jaccard similarity index with increasing SeqID and SeqLC, however, the number of clusters is also increasing monotonously. We find that the SeqID influence is higher than the SeqLC effect on the number of detected clusters (by a factor of 1.5–2; see Additional file 2: Tab. S2).

**Fig. 3:**
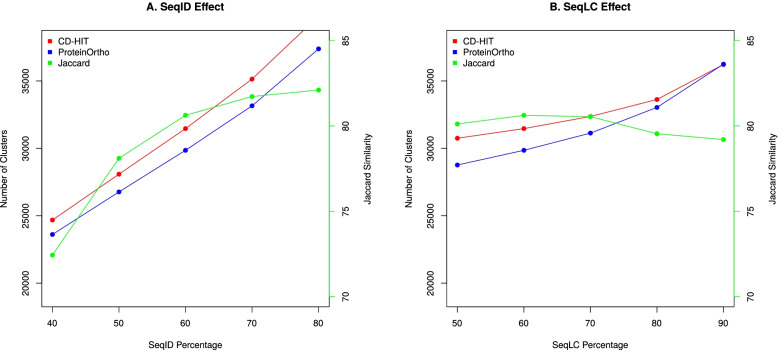
The SeqID effect (**A**) at SeqLC=60% and SeqLC effect (**B**) at SeqID=60%. The *x*-axis represents the SeqID percentage (**A**) or SeqLC percentage (**B**) to evaluate the effect of SeqID and SeqLC, respectively. The red and blue lines show the number of clusters (left *y*-axis) detected in CD-HIT and ProteinOrtho, respectively, whereas the green line represents the Jaccard similarity index as shown in the right *y*-axis

We observe a near plateau of the Jaccard similarity index for both SeqID and SeqLC at about 60% (see Fig. 3). This result essentially repeats the outcome of the *Streptococcus pyogenes* pangenome study published earlier [49]. We argue that SeqID=60% and SeqLC=60% are the optimized parameters for generating the clusters of homologous genes/proteins also in the *E. coli* case. To note, too relaxed parameters lead to smaller number of clusters albeit (i) the possible occurrence of actually non-homologous genes/proteins in the same cluster and (ii) a low concordance between the two different methods for homology assignment. At the same time, too stringent parameters create a much larger number of clusters/GFs (by essentially breaking up true homologous clusters) with no significant increase in the concordance between the two methods.

Even with the optimal choice of SeqID and SeqLC, manual analysis of selected GFs shows some clusters being split into two or more groups with the sequentially more distant members forming independent GFs. Therefore, we introduced a re-clustering phase to reduce the scale of this problem. We selected longest sequences from each cluster as representative leads and subjected them to re-clustering by CD-HIT with parameters SeqID=40% and SeqLC=50%; however, with the additional criterion that the merged clusters must share the same PFAM domains as illustrated in Fig. 2. Notably, the re-clustering approach leads to an increase of the Jaccard similarity index from 80.62% (Additional file 2: Tab. S1) to 87.97% (Table 1), as well as a reduction of the pangenome size from ~30,000 gene families to ~25,000 gene families.

**Table 1:** Pangenome profile in 1,324 *E. coli* identified based on CD-HIT and ProteinOrtho. The Jaccard index measures the similarity between the two methods. The softcore genome is defined as the set of clusters of homologous genes, which exist in at least 95% of the genomes

Methods	PanGenome	Core Genome	Softcore Genome	Singletons
CD-HIT	25,420	425	3057	5654
ProteinOrtho	24,889	427	3056	5568
**Jaccard Index**	**87.97%**	**95.41%**	**95.49%**	**93.28%**

### Pangenome profile of 1324 *E. coli* complete genomes

Table 1 shows the summary of the pangenome, core genome, and softcore genome sizes of the 1324 *E. coli* strains as calculated with the two methods CD-HIT and ProteinOrtho. We have provided the pangenome matrix with Supplementary file 2 for the CD-HIT and Supplementary file 3 for the ProteinOrtho method, respectively (in zip package Additional file 3).

**Fig. 4:**
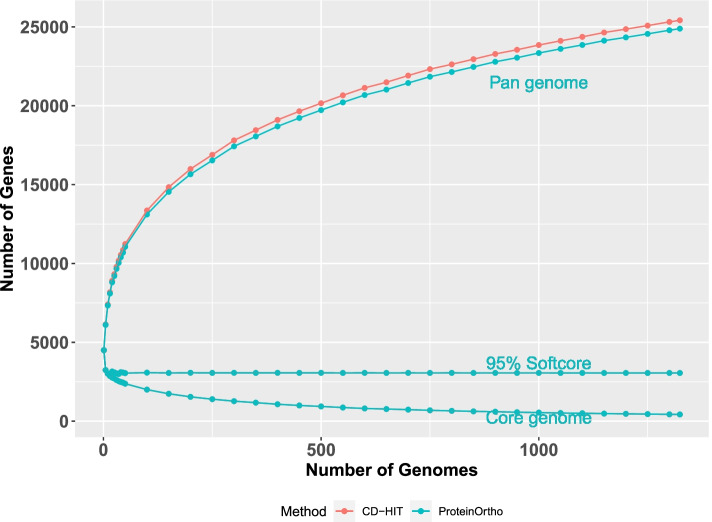
Pangenome plot of *E. coli* genomes across different number of genomes used for pangenome construction. Along the *x*-axis, we indicate the number of genomes used for pangenome construction and the *y*-axis shows the number of identified gene families. Each small filled circle specifies the average number of genes identified across 100 random permutations of randomly selected genomes. The number of genomes tested range from 5, 10... 50, 100, 150, 200, 250...1200, 1250, 1300, to 1324. The red and blue lines represent the CD-HIT and ProteinOrtho method results, respectively. The pangenome, core genome and softcore genome lines are shown accordingly. The procedures using CD-HIT or ProteinOrtho give the same or very similar softcore and core genome sizes and, therefore, the respective two curves overlap. Tettelin et al. [41] demonstrated that the number *N* of distinct gene families (= pangenome size) computed from *n* genomes can be estimated with a power law-type model (Heap’s Law) as *N* = *kn*^(1 − *α*)^with curve fitting constants *k* and *α*. The pangenome is said to be open (infinitely growing with *n*) if *α* < 1. Otherwise (*α* ≥ 1), it is a closed pangenome. This pangenome seems open (if computed with ProteinOrtho data, *α* ~ 0.7439 and *k* = 4206; for the CD-HIT curve, *α* = 0.7521 and *k* = 4221)

The concordance of the pangenome clusters between the two methods is at least 87%; i.e., there are at least 87% common clusters among them. We have ~25,000 GFs in the *E. coli* pangenome, ~420 GFs are in the core genome and ~3050 GFs belong to the softcore genome. Figure 4 illustrates how the pangenome, the core genome size, and the softcore genome (GFs in ≥95% of strains) sizes change as the number of genomes *n* increases. As we can clearly see, the pangenome size grows monotonously without visible signs of saturation.

To quantify the growth trend, we approximated the curve with Heap’s law (see legend in Fig. 4). When the *n*th genome is added, as a trend, further genes with a number proportional to *n*^−*α*^ (with *α* being about 0.75 and clearly smaller than one) complement the pangenome. Notably, *α* for a 4071 genome ST131 set was found to be in a very similar range [57]. Thus, *E. coli* appears to have an open pangenome. At the same time, the core genome size decreases to a ridiculously small number that is hardly sufficient to make up a surviving *E. coli* cell. The number of absolutely essential genes in *E. coli* is estimated to be in the range of a few hundred (303 as reported by Baba et al.) [58, 59]. The number of essential genes remains hotly debated as gene interactions and specifics of experimental assays cannot be ignored. Actually, the value that the core genome size in Fig. 4, if extrapolated as further genomes get added, is approaching this number.

### The softcore genome is stable and consistent with at least 100 genomes in the pangenome analysis

**Fig. 5:**
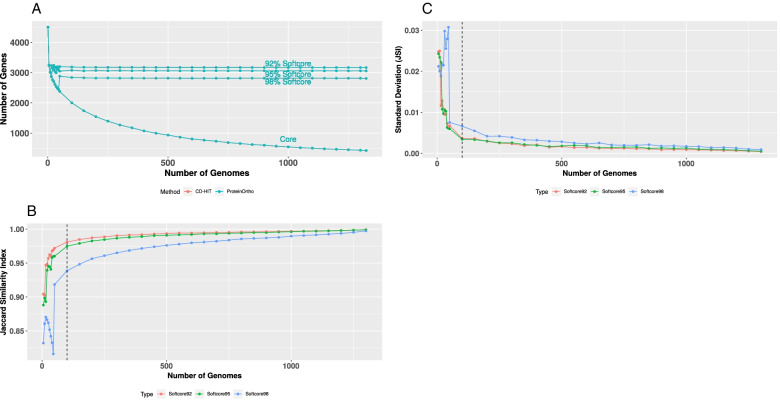
**A** Evaluation of softcore genome definition across different number of genomes. The CD-HIT and ProteinOrtho methods give the same or very similar results and, therefore, the lines overlap. The evaluation is performed for three definitions of the softcore genome, i.e., 92, 95, and 98%. **B, C** The mean and standard deviation of the Jaccard similarity index comparing the softcore genome at a specific number of genomes from the total set of genomes (i.e., 1324 genomes). The high mean Jaccard similarity index together with a low standard deviation suggests the stability and consistency of the softcore genome at different definition levels. The vertical black dashed line marks the number of genomes equal to 100

The softcore genome (defined as set of GFs in at least 95% of all genomes) size is ~3050, which is consistent with the value from a previous study (~3000) using just 186 genomes [43, 60]. Interestingly, we find that the softcore genome size is stable once a sufficient number of sufficiently diverse genomes (>100) has been included into the pangenome analysis (Fig. 4). To test the robustness of the observation and the influence of parametrization, we varied the definition of the softcore genome as shown in Fig. 5A (exploring the thresholds 92% and 98% in addition to the standard 95%). To our surprise, we obtained stable softcore genome sizes of ~3200 GFs (for 92%) and ~2800 GFs (for 98%) as long as the number of sufficiently diverse genomes in the pangenome analysis is larger than 100.

Nonetheless, similarity in size does not necessarily mean similar members of GFs. Therefore, it is important to evaluate if the stability in the softcore genome size reflects consistency of the softcore genome clusters as well, i.e., the same or similar members of gene families are identified independently of the number of genomes used to generate the pangenome. We calculated the softcore genome clusters 100 times for random selections of 5, 10, 15… 50, 100, 150, 200… 1300 genomes and determined the average (Fig. 5B) and the standard deviation (Fig. 5C) of the Jaccard similarity index at each point. Subsequently, we evaluated the Jaccard similarity index between the softcore genome clusters at different genome sizes to the softcore genome clusters with 1324 genomes. Evidently, we can see that, for the 92% and the 95% thresholds, the softcore genome is not only stable with regard to total size but also consistently determines almost the same set of GFs when we have at least diverse 100 genomes in the pangenome analysis. In the case of the 98% threshold for the softcore genome generation, more genomes (>1000) are needed to achieve similar levels of numbers of related GFs in the softcore genomes.

Further, if we compare the softcore genome cluster sets calculated with 100 genomes (for the 92% and 95% thresholds for softcore genome generation) with that obtained from the full set of 1324 genomes, the two softcore genomes have about 98% clusters in common.

The stability and consistency of the softcore genome (i.e., the stable size and GF composition regardless of the number of genomes included) happen apparently not by chance. A previous study with 48 *E. coli* genomes [60] experimented with the notion of a “percent pangenome” based on the percentage of genomes sharing the GFs. The authors note that there is a trend for saturation (for example, for the 50% pangenome) despite any increase of the number of genomes included into the pangenome. In this context, it is also notable that the distribution of functional categories among COGs [61] found in the GFs in the *E. coli* softcore genome is essentially the same as that of the functional attributes of COGs associated with the two *E. coli* genomes *E.* O157:H7 str. Sakai and *E. coli* str. K-12 substr. MG1655 (see Additional file 1: Fig. S5). Our findings suggest that the softcore genome (with a 92–95% generation threshold but not with higher thresholds) could be used to define the genome of a bacterial species (particularly that of *E. coli*) listing the critically relevant, evolutionarily most conserved, biologically most important classes of GFs.

### The accessory genome reveals specific distinct gene family clusters in different sequence types and phylogroups of *E. coli*

The STs and phylogroups of all *E. coli* strains are available in Supplementary file 1 (as part of zip package Additional file 3). The pangenome matrix provides the opportunity to explore the molecular characteristics of different sequence types or phylogroups of *E. coli* genomes. As there are many rare STs of *E. coli* genomes, we have focused only on the 21 STs with at least 10 genomes. This gives a total of 674 genomes and 6244 GFs (in at least 10 genomes and at most 640 genomes) for analysis. The purpose is to evaluate the most informative GFs in this accessory genome.

**Fig. 6:**
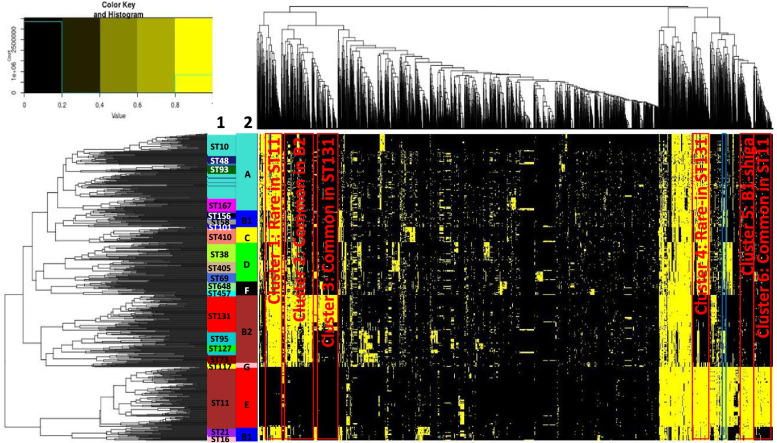
Heatmap profiling of GFs for frequently observed sequence types. Heatmap profiles have been derived by unsupervised clustering of 674 *E. coli* genomes with common sequence types (STs), i.e., at least 10 genomes in each ST. There are 6244 GFs included in the analysis with presence in at least 10 genomes and at most 640 genomes, respectively. The yellow color represents present and black color represents absent GFs (column) in the genome (row), respectively. The ST (1) and phylogroups (2) are labelled accordingly. The dendrograms for genomes and GFs are shown in rows and columns, respectively. The six most distinguishing patterns are highlighted in red boxes representing Cluster 1 (Rare in ST11), Cluster 2 (Common in B2), Cluster 3 (Common in ST131), Cluster 4 (Rare in ST131), Cluster 5 (B1-shiga), and Cluster 6 (Common in ST11). The blue box represents the cluster with T6SS-2 in ST11

Figure 6 shows the heatmap profile (presence/absence matrix) of these 674 genomes with unsupervised clustering at the genome and GFs level. We annotated the genomes with its corresponding ST and phylogroup, respectively. At the genome level, it can be clearly seen that the pangenome profile correlates well with the STs as well as the phylogroups. In fact, sequence types are associated with phylogroups without any ambiguity. While the different phylogroups can be distinguished from each other clearly, interestingly, the phylogroup B1 has two distinctive clusters, i.e., one that groups together with phylogroups A and C, and the other that clusters together with phylogroup E. The former B1 cluster includes ST58, ST101, and ST156, whereas the latter comprises ST16 and ST21. We find that the latter B1 cluster carries Shiga toxin genes, whereas the former one does not have the Shiga toxin. This suggests that the strains in the B1-non shiga cluster are more likely to be of low virulence (likely nonpathogenic). At the same time, the B1 strains with the Shiga toxin are more likely to be of high virulence similarly to ST11 *E. coli* from phylogroup E.

At the gene families’ level, several distinct GF clusters specific for certain groups of phylogroups or sequence types are clearly recognizable at the background of scattered minor differences (Fig. 6). We highlight the six most obvious GF clusters distinguishing sequence types and phylogroups:

1. Cluster 1 (rare in ST11);
2. Cluster 2 (common in B2);
3. Cluster 3 (common in ST131);
4. Cluster 4 (rare in ST131);
5. Cluster 5 (B1-shiga); and
6. Cluster 6 (common in ST11).

The list of GFs for each group is provided in Supplementary file 4 (as part of zip package Additional file 3). Further below, we analyze the biological implications that can be derived from the functional annotations of those genes, especially for three types of *E. coli*, particularly the *E. coli* ST11 group (basically O157 EHEC), the *E. coli* ST131 strains, and the phylogroup B1 *E. coli*.

### Unique characteristics of ST131 *E. coli*

ST131 *E. coli*, one of the most important *E. coli* clonal group, which belongs to phylogroup B2, has risen to prominence in recent years due to its prevalence among the ExPEC *E. coli* including UTIs and bloodstream infection as well as its multidrug-resistant profile [12, 13, 62–65]. It can be easily seen in Fig. 6 that there are two distinct groups of gene families that characterize *E. coli* ST131—Cluster 3, a cluster of genes, which are common in ST131 genomes and rare or completely missing in almost all other phylogroups, and Cluster 4, a cluster of genes, which are rare in ST131 genomes, but common in almost all other genomes. Unfortunately, a considerable number especially of Cluster 3 genes are incompletely or not at all functionally annotated. In this section, we focus on the genes with well described function. In the next section, we will dive into the yet functionally uncharacterized part.

The analysis of the distribution of COG functional categories [61] reveals the enrichment of cluster 3 with mobilome-related genes (Additional file 1: Fig. S6A). Their relative occurrence in the gene set is at least 8-fold higher than in the functional category distribution for the COGs for the two *E. coli* reference genomes (Additional file 1: Fig. S5A) and more than 30-fold higher compared to that in the softcore genome computed in this work (Additional file 1: Fig. S5B). This observation is also in sharp contrast to the occurrence of mobilome genes in Cluster 1 (more than 30 times lower than in Cluster 3, see Additional file 1: Fig. S 6B). Thus, expansion of the mobilome was one of the critical innovations of ST131 in evolution compared to other *E. coli* strains.

There are several important annotated genes in Cluster 3 that help understanding the nature of ST131. For example, the gene *wzx* is the O25 family O-antigen flippase. This explains why the ST131 strains are dominated by the O25 serotype, probably, a sampling artifact due to the biased selection of strains for genome sequencing.

The gene *sat* (secreted autotransporter toxin) is a known virulence factor implicated in uropathogenesis [66]. It belongs to the SPATE gene family (serine protease autotransporters of enterobacteriaceae) that includes multiple virulence factors involved in bloodstream infection [67]. We have noted that *sat* also exists in 30% of the phylogroup D strains, but it is rare in A and F. It is completely missing in phylogroup E.

Whereas the distribution of functional categories in Cluster 4 (Additional file 1: Fig. S6B) is very similar to that of the COGs for reference genomes (Additional file 1: Fig. S5A) and for the softcore genome (Additional file 1: Fig. S5B), the suspicious relative absence of metabolome-related genes is another distinguishing feature of ST131. The following five GFs are in Cluster 4:

1. The cluster of *frv* genes,
2. The cluster of *hca* genes,
3. The cluster of *pao* genes,
4. The cluster of *puu* genes and
5. The cluster of *lsr* genes.

Briefly, *frvA* from the cluster of *frv* genes has been shown to be sensitive to iron intoxication [68]. The *hca* cluster is involved in the catabolism of different phenylpropanoid compounds [69] and, hence, affects the tolerance to the living environment for ST131. The *pao* gene cluster has been thought to play a role in detoxifying aromatic aldehydes [70]. The *puu* gene cluster is part of the putrescine utilization pathway genes, which means that lacking this gene suggests inability of utilizing putrescine for growth [71]. The *lsr* operon has been suggested to affect overall strain fitness [72]. Its induction increases the pathogenicity of APEC [73] whereas deletion of *lsr* operon leads to reduction of virulence.

### Synteny analysis of common gene families in ST131 *E. coli* reveals the presence of full intact prophage with tailocin structure

In the previous section, we have discussed some well-annotated common and rare GFs of *E. coli* ST131 strains. There are many genes in cluster 3 with cryptic or absent functional description. As a step towards their functional characterization, we performed a synteny analysis among them and we identified two synteny clusters that are highly conserved across the ST131 *E. coli* genome. The two clusters (s1-ST131 and s2-ST131) are shown in Additional file 2: Tab. S3. Both clusters have a length of approximately 23 kbp involving 30 genes and 33 genes, respectively. Next, we investigated the DNA sequences of the two clusters by evaluating (1) the specificity of the synteny regions for *E. coli* ST131 and (2) the sequence homology to other species (excluding *E. coli*).

To investigate the specificity of the clusters, the DNA sequences of each cluster are searched with blastn against the ST131 and non-ST131 genomes, respectively. Additional file 1: Fig. S7 shows clearly that the two clusters are highly conserved in the ST131 *E. coli* genomes. There are 13 hits to non-ST131 genomes with >90% sequence coverage for the first cluster (s1-ST131), whereas there is only a single hit to non-ST131 genomes for the second, longer cluster (s2-ST131). We find that 68 out of the 79 *E. coli* ST131 genomes carry the s2-ST131 synteny region. Only a single non-ST131 strain, the singleton ST2279, has it as part of its genome. Further investigation shows that the difference in sequence typing between ST131 and ST2279 is due to just a single-nucleotide difference at position 263 bp of the gene *purA* (ST131 carries *purA_8* allele and ST2279 carries *purA_28* with the mutation *purA.*263 G>T). Thus, ST2279 is rather close to the ST131 group if not just a misclassified case due to a sequencing inaccuracy.

To find homologous sequences in other species, we use NCBI blastn to the non-redundant database excluding *E. coli* genomes. The top 20 hits are shown in Additional file 1: Fig. S8 and Additional file 1: Fig. S9 for s1-ST131 and s2-ST131, respectively. With high sequence similarity and coverage, the synteny region s1-ST131 hits into the genomes of several pathogens such as *Klebsiella* species, which share a similar living environment as *E. coli*. It also has very high similarity to *Myoviridae sp.*, which is a class of bacteriophages (Accession ID: BK037528.1).

In contrast, s2-ST131 has 100% identity with and coverage by a bacteriophage sequence (Accession ID: BK034715.1). Both bacteriophages BK037528.1 and BK034715.1 were recently reported [74] as the viral components from microbiome samples. This provides experimental evidence that these prophage regions in the genome of ST131 strains might be expressed during SOS response, a complex bacterial reaction to DNA damage with cell cycle arrest, DNA repair, and induced mutagenesis [75].

Next, we investigated the protein sequences for each of the genes within the s1-ST131 and s2-ST131 synteny clusters as described in the “Methods” section. The protein sequences were submitted to HHPRED, blastp, and ANNOTATOR and manual analysis of results was performed. We observed that some of the protein sequences have obvious similarity to pyocin R2 components of *Pseudomonas aeruginosa* PAO1 [76]. Given this clue, we further annotated all the genes in s1-ST131 and s2-ST131 relative to proteins in *Pseudomonas aeruginosa* PAO1 as shown in Additional file 2: Tab. S4 and Additional file 2: Tab. S5, respectively. Genes that remained unmapped to *Pseudomonas aeruginosa* PAO1 were annotated with the most significant hits from the in-house sequence analysis (see the “Methods” section) accordingly.

Interestingly, some of the s1-ST131 genes map only to a sub-structure of pyocin R2 in *P. aeruginosa* PAO1 (a tailocin [77]), whereas the s2-ST131 genes can be aligned to the complete structure of pyocin R2 with the exact same order of genes in the operon. The actual protein sequence identity is not high (from 24 to 47%) but the fold- and function-critical sequence profiles of the 13 of the 14 components of the tailocin nanomachine are detected with search tools such as blastp and HHPRED.

It is worth noting that there might be a potential annotation error in the synteny region s2-ST131 for the remaining 14th gene (late control gene: SY51_RS10535 of GCF_000931565.1) between the loci for GF_6212 and GF_13723 (Additional file 2: Tab. S6). In RefSeq, it is annotated as a predicted pseudogene due to a frameshift. In fact, this genome region is 100% identical with the late control protein (accession DAS35886.1). In view of this, we added DAS35886.1 between GF_6212 and GF_13723. This finalizes the mapping of the first 14 genes of s2-ST131 to the complete structure of pyocin R2 of *P. aeruginosa* PAO1. It would be interesting to see if this interpretation can be experimentally validated.

For the next 19 genes, manual annotation suggests that the next 10 genes seem to code for the capsid head of bacteriophage and, finally, the rest of genes code for lysis-related proteins (Additional file 1: Fig. S10 and Additional file 2: Tab. S5).

The striking similarity of synteny regions s1-ST131 and s2-ST131 to a bacteriophage suggests integration into the *E. coli* ST131 genome of prophage after lysogenic infection. Notably, these two regions were previously reported as potential mobile genetic elements as prophage 2 (similar to s1-ST131) and prophage 5 (similar to s2-ST131) [78, 79]. We used PHASTER [80] to evaluate the DNA sequences for the presence of functional bacteriophage sequences. Synteny region s1-ST131 has an incomplete prophage structure with score of 30. In contrast, synteny region s2-ST131 has intact prophage structure and reaches the maximum score of 150. Taken together, the results suggest that s1-ST131 appears a prophage remnant, whereas s2-ST131 seems a functional prophage. As we have shown above, this synteny region encodes a prophage with its tail resembling the tailocin structure that has been demonstrated to be functional as killer weapon [77].

### Variation of bacterial secretion system and iron acquisition system in *E. coli* ST11 and B2 groups

*Escherichia coli* ST11 has the pathotype EHEC with the O157 serotype. Its distinct molecular capabilities are characterized by three GF clusters: Cluster 1 (rare in ST11), Cluster 2 (common in phylogroup B2 but very rare in ST11), and Cluster 6 (common in ST11). Given the subsets of well-annotated genes, we find that genic variations in *E. coli* ST11 affect the bacterial secretion systems (type II secretion system (T2SS), type IV secretion system (T4SS) and type VI secretion system (T6SS)) as well as the iron acquisition system.

All *E. coli* ST11 strains (except two: *E. coli* O157 strain A1 Ain / GCF_008462425.1 and *E. coli* strain M7638 / GCF_009432795.1) carry plasmids with genes for the type II secretion system (T2SS) as part of GF Cluster 6. The T2SS operon in ST11 is called *etp* (EHEC type II secretion pathway) [81], whereas in other non-ST11 *E. coli*, the T2SS is of a different type and the operon is identified as *gsp* (general secretory pathway). T2SS has been shown to contribute to bacterial pathogenicity [82], either through delivering toxins to the mammalian host [83] or by helping the bacteria to adapt to the host environment [84, 85]. Concordantly, the T2SS *etp* gene clusters have also been shown to be important for bacterial adaptation to its environmental niche [86, 87].

The Type IV secretion system (T4SS) is typically not found in *E. coli* ST11 but is common in phylogroup B2 and sporadically exists in other phylogroups (Cluster 2). The bacterial T4SS is a very diverse and versatile system, which serves a variety of purposes by secreting macromolecules (either DNA or proteins or protein-DNA complexes) into prokaryotic or eukaryotic cells to facilitate their proliferation and survival [88, 89]. It also plays an important role in bacterial evolution as conjugation system [90]. There are three subfamilies of T4SS in prokaryotes, i.e., (i) conjugation systems; (ii) effector translocator systems; and (iii) DNA release or update systems [91]. While different types of T4SS exist in our *E. coli* genomes, we have noticed that a variant of T4SS (Type IV conjugative transfer proteins, from the *tra* gene clusters as shown in Supplementary file 4 (part of zip package Additional file 3)) is common in phylogroup B2. It has been suggested that the T4SS conjugative system represents a selective advantage in disseminating antibiotic resistance genes [89]. Coincidentally, we have observed a wide spread of antibiotic resistance genes present in the phylogroup B2, particularly in *E. coli* ST131; yet, the presence of antibiotic resistance genes in the ST11 *E. coli* is limited (Supplementary files 5 and 6 as part of the zip package Additional file 3).

There is sequence type and phylogroup variation with regard to T6SS among the *E. coli* genomes. Three variants of T6SS have been reported in *E. coli* (T6SS-1, T6SS-2, and T6SS-3 [92]). T6SS-1 and T6SS-3 are known to play a role in antibacterial activity whereas T6SS-2 is important for pathogenesis. Interestingly, we have observed that T6SS-1 is common in the phylogroup B2 (cluster 2), particularly in ST131 but it is very rare in the ST11 *E. coli*. In contrast, the T6SS-2 (highlighted in blue box in the heatmap of Fig. 6) is very common in ST11, in B1 shiga, it also appears sporadically in other groups. The toxins or effectors secreted by T6SS are very diverse reflecting the T6SS activity and the complexity of the T6SS roles in *E. coli* [92, 93].

We have also noticed a manganese catalase family protein (RefSeq ECs_1652, GeneID 913226, UniProtKB Q8XDQ1) that exists in almost all of the ST11 genomes. A novel effector *katN*, which is a Mn-containing catalase, has been reported by Wan et al. [94] to be secreted by T6SS in EHEC and to be important for surviving macrophage phagocytosis.

Differences in iron acquisition system have also been detected by heatmap analysis (Fig. 6). Particularly for the GF cluster that is common in phylogroup B2 (Cluster 2), we have seen an enrichment of yersiniabactin siderophore, aerobactin siderophore, and iron/manganese ABC transporter genes. Yet, these genes are missing in the *E. coli* ST11. As an alternative, the *chu* operon for heme uptake is present. The *chu* operon is not specific to ST11, it exists in phylogroup B2 as well. This suggests that, while the phylogroup B2 has a wide variety of iron acquisition systems (with implications for its improved survival capability), the iron acquisition system in ST11 *E. coli* seems to be limited or narrow (in agreement with [34]).

### Synteny cluster analysis of common gene families in ST11 *E. coli* reveals a potential pathogenicity island

With the same approach as with the ST131 *E. coli* sequences, we have also performed synteny analysis on the ST11 *E. coli* genomes. We identified two synteny clusters (s1-ST11 with 16 genes and 19 kbp length and s2-ST11 with 18 genes and 15 kbp length; see Additional file 2: Tab. S6).

Similarly, we investigated the DNA sequences of the two clusters according to their (i) specificity among *E. coli* strains and (ii) sequence homology to other species (excluding *E. coli*). Additional file 1: Fig. S11 (Additional file 1) shows that the two clusters are highly conserved and prevalent across ST11 *E. coli* genomes. However, 34 non-ST11 genomes contain an s1-ST11 cluster and 64 non-ST11 genomes comprise an s2-ST11 synteny region. Most of them belong to other sequence types of phylogroup E. But a substantial fraction of the non-ST11 genomes are members of phylogroup D. All these non-ST11 sequence types have few genome representatives (i.e., < 10 genomes) and, therefore, were not included in our exploratory analysis of the 674 *E. coli* genomes.

Next, we use NCBI blastn to query these two synteny clusters against the non-redundant database excluding *E. coli* genomes. The top 20 hits are shown separately for s1-ST11 (Additional file 1: Fig. S12) and for s2-ST11 (Additional file 1: Fig. S13). The synteny cluster s1-ST11 hits best to sequences from *Enterobacter mori*, *Enterobacter cloacae*, and *Enterobacter hormaechei*. The hits, however, only cover 40% of the sequence with ~77% identity. While *Enterobacter mori* has been commonly associated with plant pathogens [95], the other two bacteria are known from nosocomial infections [96–99].

The second cluster s2-ST11 has more than 95% identity to a chromosomal segment of *Escherichia fergusonii* with 100% coverage. *E. fergusonii* is closely related to *E. coli* and has been reported to cause hemolytic urine syndrome [100]. *E. fergusonii* has been isolated from the feces of animals [101] as well as wounds and urinary tracts of human [102].

The synteny cluster s1-ST11 has been predicted to be involved in bacterial pathogenesis and lipoprotein metabolism. The presence of lipid metabolism genes in this cluster (subset of genes ECs_1284 to ECs_1289; actually part of a biosynthetic gene cluster—see below) suggests the ability of *E. coli* ST11 to produce fatty-acid containing molecules [103]. ECs_1282 gene (hemagglutinin/hemolysin-related protein) in the s1-ST11 cluster has also been suggested to be a virulence factor in multiple studies [104–106].

The s2-ST11 synteny cluster ranges from ECs_4324 to ECs_4341 and contains lipoprotein and fatty-acid biosynthesis systems. Both genomic regions called here s1-ST11 and s2-ST11 have been reported to be part of S-loop #71 and S-loop #225, respectively [107]. They are induced in the *E. coli* O157 Sakai strain during the spinach root interaction [86], which suggests their importance during early interaction of *E. coli* ST11 (O157) with the fresh-produce plant.

Biosynthetic cluster analysis (with antiSMASH 6.0 [108]) reveals that an aryl polyene (APE) biosynthetic gene is part of s1-ST11 (Additional file 1: Fig. S14) and the APE gene cluster (BCG0000836) is present in s2-ST11 (Additional file 1: Fig. S15). A recent study [109] has shown that APE increases the fitness of bacteria populations by protecting them from oxidative stress and contributing towards biofilm formation. Apparently, the two clusters are important for the survival of *E. coli* ST11 as a foodborne pathogen.

### *Escherichia coli* phylogroup B1 can be differentiated into groups with regard to the pathogenicity mechanism

Based on the heatmap profile, we observe that *E. coli* phylogroup B1 is split into two groups, i.e., one is together with phylogroups A and C and the other clusters with phylogroup E. Notably, the latter group of strains (1) carries the shiga toxin genes suggesting their potential pathogenicity (“shiga B1” and “non-shiga B1” strains).

In our large genome collection, we find that three further gene groups are characteristic for shiga B1 strains (Shiga toxin-producing *E. coli* (STEC)), namely (2) the T3SS LEE cluster of genes [110], (3) the cluster of *ter* (tellurium resistance) genes [111], and (4) the cluster of *ure* (urease) genes [112] in agreement with the literature based on much smaller genome collections. LEE-positive Shiga toxin *E. coli* strains are known to cause bloody diarrhea with possibly life threatening hemolytic uremic syndrome (HUS) [113]. We find that majority of these strains belong to the O111:H8 and O26:H11 serotypes (non-O157 EHEC genomes).

### GF coincidence analysis provides insight into pathogenic effects of GFs significantly associated with the s1-ST11 and s2-ST131 clusters

The accessory genome matrix with 6244 GFs from 674 genomes (as described above) was used for coincidence analysis with CoinFinder [114]. The program excluded 2299 GFs due to low frequency as they are presented in less than 5% of the 674 genomes. The remaining 3945 GFs are evaluated for pairwise association. Additional file 1: Figure S16 shows the distribution of all potential pairwise association *P*-values. If we assume the ad hoc selected *P*-value ≤ 10^−20^ as significance threshold, we still have 233,483 significant pairwise associations for 3338 GFs (Supplementary file 8 in the zip package Additional file 3). Most of the GFs have fewer than 50 associated GFs (through pairwise association); however, quite a substantial number of the GFs have more than 300 associated GFs (Additional file 1: Fig. S17).

The comprehensive analysis of this GF coincidence data will be presented elsewhere. Here, we focus on the GFs associated to s2-ST131 and s1-ST11. As expected, we find the GFs in s2-ST131 being associated to those in s1-ST131 and vice versa. There are about 360 GFs associated to GFs in s2-ST131 and approximately 590 GFs associated to GFs in s1-ST11 as shown in Additional file 2: Tab. S7 and Additional file 2: Tab. S8 respectively. We performed synteny cluster analysis of the associated GFs to investigate if there is any potential operon or cluster of genes that is associated to s2-ST131 and/or s1-ST11. Interestingly, we observed that there is a cluster of flagellar genes (closely related to type III secretion system or T3SS) as well as another, type VI secretion system (T6SS) gene cluster associated to s2-ST131 (Additional file 2: Tab. S9). Similarly, we have also observed that T3SS, a tellurium resistance gene cluster as well as prophage clusters are associated to s1-ST11 (Additional file 2: Tab. S10). To note, T3SS [115] and T6SS [92, 93] are known to be associated with pathogenicity.

### Likelihood of pathogenicity is correlated to number of prophages instead of the antibiogram

Next, we investigated if there is any correlation between the virulence category (“likely nonpathogenic,” “likely virulent,” “highly virulent,” and “extremely virulent”) with the number of prophages contained in the genome as well as the likelihood of antibiotic resistance as defined by antibiogram. The antibiograms of 24 AMR targets for the 1324 genomes are provided in Supplementary file 6, whereas the virulence factor matrix is given in Supplementary file 7 (both in the zip package Additional file 3).

**Fig. 7:**
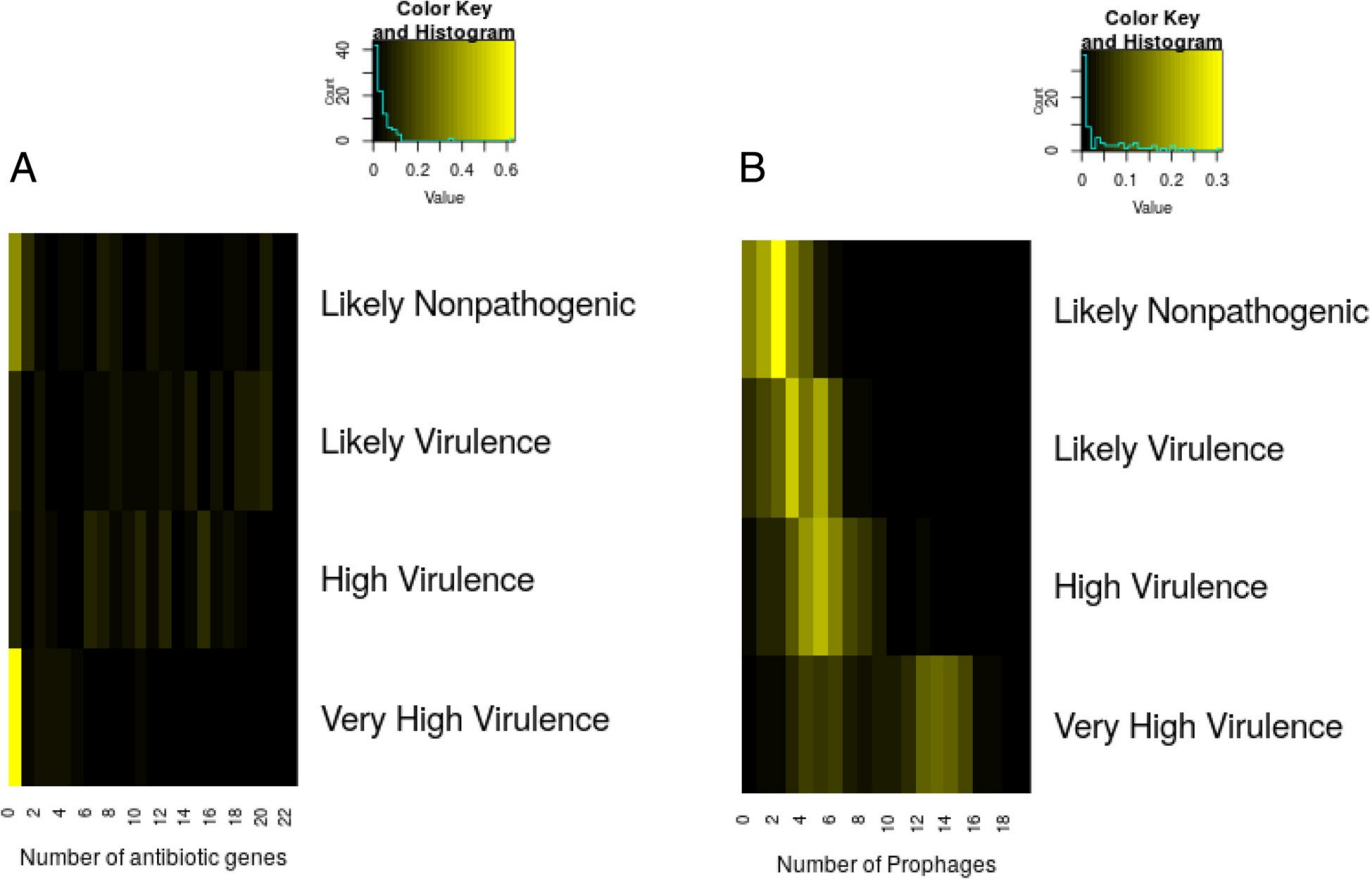
Virulence as function of the antibiogram and of the number of prophages. The relationship of virulence category to **A** antibiogram (number of antibiotic genes’ presence in the genome) and **B** number of prophages. The relationship is shown as a heatmap profile with the brightness of the yellow color representing the proportion of genomes with that criteria. Black color indicates absence of genomes, whereas the brightest yellow represents the highest proportion of genomes in that category

Figure 7 shows the relationship between virulence category to the number of antibiotic resistance genes as well as the number of incorporated intact prophages. It can be clearly seen that there is no relationship between the likelihood of multiple drug resistance with the virulence category. Even the genomes with very high virulence category do not necessarily have a high number of antibiotic resistance genes. In contrast, the number of intact prophages is correlated to the virulence category. There is a tendency that genomes with higher number of intact prophages have a higher number of virulence genes and are more likely to be virulent. This is expected because it is common for prophages to carry virulence factors in their DNA.

## Discussion

First studied in 1844, *E. coli* has become one of the most intensively analyzed model organisms. However, its diversity and versatility in different environments and ecological niches, its usage as laboratory and biotechnology work horse, and its relevance in animal and human pathogenicity suggest that research on *E. coli* has value far beyond its role as a model organism [1].

Both significance of the *E. coli* system as well as the wide and growing availability of relevant sequence data enabled a plethora of previous work in this field [34, 39–46]. As the computational load for the gene family computation and genome comparison becomes easily overwhelming with larger genome numbers, various shortcuts have been explored. Methodical restrictions (such as ad hoc selected values for SeqLC and SeqID for gene/protein homology criteria instead of scanning a range and finding optimized numbers, ad hoc thresholds for definitions of softcore and accessory genomes, etc. [116, 117]) or approximations (such as genomic distances based on *k*-mer patterns [118–120]) were regularly applied. Clearly, incompleteness of many genomes in the dataset will affect size of the pangenome and its computed constituent subsets.

Despite the limitations, several of those literature reports arrived at notable conclusions. An approximated, *k*-mer pattern-based genomic distance was sufficient to recreate the known phylogroup classification with a set of 10,667 mostly incomplete genomes and to suggest an evolutionary path of *E. coli* subtype differentiation [120]. Decano et al. [57] studied genomes from 4071 ST131 isolates (most of them incompletely sequenced) and classified them into three genetically distinct clades A, B, and C (with three subclades). A GWAS study based on a pangenome matrix derived from *E. coli* genomes (extracted from 309 diseased and 234 asymptomatic carrier chicken) identified disease-associated variations in 143 *E. coli* genes [117]. Interestingly, it was proposed to use the pangenome matrix for assessing the coincidence rate of GF presence in genomes and to explore potential biologically significant interactions between genes [114].

In this work, we have analyzed 1324 *E. coli* complete genomes from the NCBI Refseq database. This work is aimed at exploring this genome set from three perspectives. First, we wanted to study the pangenome profile and to derive optimal parameters for its development. Second, we wished to find biomolecular characteristics for sequence types and phylogroups. Third, we wanted to explore their relevance for pathogenicity. Subsequently, this study provides us with a better understanding of *E. coli* as a bacterial species and what are the still missing, unknown elements.

We have built the *E. coli* pangenome according to Fig. 2. Using the optimized parameters of SeqID=60% and SeqLC=60%, we estimated the pangenome, core genome, and softcore (95%) genome size to be ~25,000, ~400, and ~3000 gene families (GF), respectively. As pangenome size and core genome size are highly dependent on the number of genomes used, the softcore genome (defined as the GF presence in at least 95% of the genomes) is shown to be the desired representation of the species-critical genes in *E. coli*. The softcore genome is demonstrated to be stable and consistent when at least 100 sufficiently diverse genomes are included in the analysis (Fig. 5). Mapping of the softcore genomes onto the COG database reference shows that the distributions of functional COG categories are similar (Additional file 1: Fig. S5), which suggests that the softcore genome is indeed a good representation of essential genes in a bacterial species.

Notably, the pangenome size is under environmental and phylogenetic constraints [45]. With ever more *E. coli* strains from new habitats and host organisms getting sequenced, the pangenome is poised to grow further. The complete pangenome data (including the classification of gene families from all genomes studied) has been made available in the public domain for further study by the scientific community.

The pangenome matrix provides an avenue for biomolecular characterization of different *E. coli* subtypes. We focus on the most common *E. coli* sequence types with at least 10 genomes (consequently, 674 *E. coli* genomes distributed across 21 sequence types and 8 phylogroups; see Additional file 1: Fig. S1). The accessory genome (defined as the GFs present in at least 10 and at most 640 of the common *E. coli* sequence types) is used for this purpose. We identified six distinct clusters from the heatmap profile of these accessory genomes (named Cluster 1 to Cluster 6, accordingly). These six gene sets distinctly characterize three groups of strains, i.e., phylogroups B1, B2 (particularly ST131), and E (ST11), which have been described in the “Results” section. We suggest that the specific gene lists (Supplementary file 4 as part of Additional file 3) can be used as a guideline for further understanding of the specific phylogroup of interest.

Coupled with the information regarding virulence factors, antibiotic resistance genes, and prophages, bacterial pathogenicity can be understood from two different angles: virulence and survival capability (including self-defense mechanisms). Observing a virulence factor in an *E. coli* genome does not necessarily define the pathogenicity of this bacterial strain. However, it is rather the combination of multiple virulence factors and other functions that determines the pathogenicity of *E. coli* [32]. We would expect that having a larger number of virulence factors implied higher likelihood of the species being pathogenic.

Figure 2 and Additional file 1: Fig. S4 show clearly that different phylogroups or sequence types of *E. coli* have different distributions of virulence factor counts. Traditionally, it has been suggested that the phylogroups A and B1 are more prevalent among nonpathogenic *E. coli* [55, 121, 122]. However, it has also been demonstrated that the phylogroups A and B1 have very diverse pathotypes [40]. In fact, all the phylogroups manifest high diversity in the distribution of virulence factors in their strains’ genomes.

For example, we see two sub-lineages of *E. coli* in phylogroup B1 (with Shiga toxin and the other one without Shiga toxin). The number of virulence factors in the B1-shiga subgroup is much higher than in the B1-nonshiga one. This suggests that it is important not to generalize pathogenicity based on phylogroup identity alone.

On the other hand, the phylogroups B2, D, and E are enriched with genomes with generally higher number of virulence factors (Fig. 2). The phylogroups B2 and D are commonly associated with ExPEC [31, 123, 124], while phylogroup E involves the foodborne pathogen O157-serotype EHEC strain [34]. The *E. coli* strains in these phylogroups have commonly being reported to be virulent [31, 55, 125]. Concordantly, we have observed distinct variations of bacterial secretion systems (T2SS, T4SS and T6SS) among phylogroups. Bacterial secretion systems are involved in transferring toxins to host cells or for antimicrobial activity, and they are important for colonization and also for bacterial conjugation [126]. Since different pathotypes have been suggested to harbor different toxins, effectors, and infection mechanisms [16, 32, 127], the variation of these secretion systems among strains suggests different mechanisms of infection, toxin, and other effector secretion.

From the survival point of view, the bacteria’s capability to acquire nutrition, to adapt to its living environment, and to respond to external stimuli or danger [128–131] is important. Iron serves as an essential nutrient for bacteria [132, 133]. Interestingly, some infected hosts have a mechanism called nutritional immunity to limit the iron availability to the pathogen [133]. Accordingly, bacteria with multiple pathways for acquiring nutrition from their living environment have a selective advantage for their survival. The phylogroup B2 *E. coli* has multiple iron acquisition genes, such as *chu* iron heme uptake genes together with yersiniabactin as well as aerobactin siderophore genes. In contrast, the phylogroup E, particularly ST11 *E. coli*, has limited iron acquisition genes, i.e., lacking the yersiniabactin and aerobactin siderophore. This is also confirmed by a publication [34], which shows that EHEC/STEC is only enriched with *chu* iron heme uptake genes. This observation suggests that phylogroup B2 *E. coli* has a better survival capability as compared to the rest of the *E. coli* strains with the presence of multiple iron acquisition pathways. On the other hand, the pathogenicity of ST11 *E. coli* strains seems to be enhanced by the presence of aryl polyene (APE) biosynthetic gene clusters in the s1-ST11 and s2-ST11 segments as shown by antiSMASH analysis. A recent study has suggested that the APE biosynthetic gene cluster increases the survival fitness of the bacteria populations through biofilm formation [109].

In this investigation, we found that ST131 *E. coli*, a major sequence type of phylogroup B2, shows several important features that might explain why it persists in the population and it has been so successful as a pandemic *E. coli*. First, the enrichment of iron acquisition genes provides survival benefit. Second, the lack of several metabolic gene clusters (*frv*, *hca*, *pao*, *puu*, and *lsr*) leads to a leaner network. Though bacterial adaption could be achieved through loss of function [128], the full implications of this gene loss require further in-depth analysis.

Third, we have observed an enrichment of mobilome-related genes among the common genes in ST131 strains, which are missing in all other strains. These mobilome-related genes are not located in the plasmid sequence but in the chromosomes. These potentially critical genes for additional functions are most likely acquired through horizontal gene transfer [57, 134]. Synteny analysis of the common genes in ST131 reveals that some of these mobilome genes seem to form operon-like sequential stretches of DNA sequences. We identified two synteny clusters, named as s1-ST131 and s2-ST131, which are highly conserved across but distinct for ST131 *E. coli* strains (Additional file 1: Fig. S7). Prophage analysis suggested that s1-ST131 is an incomplete prophage, whereas s2-ST131 is an intact, potentially functional prophage if we apply arguments provided in the literature [135].

s2-ST131 has 100% identity to a region in BK037528.1, a recently reported bacteriophage partial genome [74]. This provides some experimental evidence that s2-ST131 appears of phage origin. Sequence analysis of proteins encoded by s2-ST131 (Additional file 2: Tab. S5) suggests that the region s2-ST131 codes for all elements of a complete bacteriophage structure (Additional file 1: Fig. S10). Thus, sequence similarity arguments suggest that the phage-tail structure resembles a complete homolog of pyocin R2 of *Pseudomonas aeruginosa* PAO1 [76] together with the presence of endolysin and holin proteins (Additional file 2: Tab. S5). It is known as tailocin [77], a phage-tail particle that is capable of killing bacteria. The similarity of pyocin R2 to the P2-like prophage suggests a close relationship of s2-ST131 to the P2 prophage [136].

The presence of prophages in bacteria has long been known in bacterial biology including its relevance to evolution, infection, and bacterial fitness [137–141]. Prophages can be induced by an SOS signal [75], which is known as spontaneous prophage induction (SPI), and it causes the lysis of host cells. The induced prophage can then function as bacteriophage infecting closely related but competitive bacteria by going through either lytic or lysogenic cycle [138, 140, 142]. In the event of lysogenic cycle, the phage DNA or potentially the host DNA can be transferred to the surrounding bacterial species [138, 142]. On the other hand, in the lytic cycle, prophage can act as a self-replicating weapon enhancing the fitness of the bacterial host population [143, 144].

Thus, the tailocin complex kills closely related bacterial strains with high specificity when the population of the producing strain is usually protected due to self-immunity [145, 146]. We think that the presence of s2-ST131 in the ST131 *E. coli* provides an advantage for these strains in the inter-bacterial competition. Therefore, in a pool of *E. coli* strains, ST131 *E. coli* may prevail over other (closely related) bacteria.

Next, we asked the question whether there is any gene family associated to the s2-ST131 and/or s1-ST11 clusters, which could allow a deepened biological interpretation of ST131 pathogenicity. The associated GFs are then investigated if they form an operon or a synteny cluster of GFs. We have observed that the s2-ST131 cluster is significantly associated with a syntenic group of flagellar genes (closely related to T3SS) and with a T6SS cluster. Similarly, s1-ST11 is jointly present with a T3SS syntenic group, a cluster of tellurium resistance genes as well as prophage clusters. All these clusters (T3SS [115], T6SS [92, 93], tellurium resistance [147], and prophages [137–141]) are known to be relevant for bacterial pathogenicity.

Finally, we investigated if the number of intact prophages has any relationship to the potential pathogenicity of the *E. coli* strain. Figure 7B shows that the number of prophages correlates with the likelihood of virulence. As prophages have the potential to carry antibiotic resistance genes and toxins [140]; therefore, *E. coli* strains with the higher number of prophages tend to be of higher virulence.

How could this competition in the human gut microbiome work? In a healthy individual, it is expected that the gut microbiome is largely colonized by commensal bacteria, which live in symbiotic relationship with the host. The symbiotic bacteria provide not only metabolic benefit, but also regulate the immune response, promote immune homeostasis, and prevent pathogen colonization in the host environment [148, 149]. As a result, the perturbation of hosts’ microbiota structure increases the risk of pathogen infection and undermines colonization resistance due to direct or indirect mechanisms [149].

Several studies have shown that, in critically ill patients, dysbiosis (disruption of the microbiota homeostasis due to an imbalance in the microflora) involves the loss of health benefits from disappearing commensal bacteria and the overgrowth by pathogenic strains [150–154]. Pathogenic *E. coli* can cause diarrhea and has also been observed in critically ill patients requiring ICU support [155, 156]. Dysbiosis observed in fecal samples from ICU patients is reflected by phylum-level composition changes with decreasing relative abundance of Firmicutes and Bacteroidetes but increasing share of Proteobacteria [151]. In the view of results shown in Fig. 7 and Additional file 1: Fig. S4 (Additional file 1), we hypothesize that, in the presence of both commensal and pathogenic *E. coli* strains found in critically ill patients, the pathogenic strains, especially those of ST131, could easily outcompete the commensal ones.

## Conclusions

This study provides the first report of applying pangenome analysis to systematically interrogate the different subtypes of *E. coli*. (1) We have built the *E. coli* pangenome from 1324 complete genomes by optimizing the parametrization with regard to the gene/protein family (GF) classification (sequence identity and sequence length coverage). This approach can be used not only for *E. coli*, but is also applicable to other bacteria. Whereas the pangenome size expands and the core genome diminishes with every new genome added, we find the softcore genome (≥95% of strains) being stable with ~3000 GFs regardless of the total number of genomes. We think that this softcore genome lists the critically relevant, evolutionarily most conserved or important classes of GFs and defines the bacterial species. (2) We have determined sequence type, serotype, phylogroup, virulence factors, antibiogram, and prophages for all genomes and studied their relationship to the strain’s pathogenicity. (3) All information collected about the pangenome GF’s and the genome properties are provided in supplementary files. Thus, this *E. coli* pangenome can serve as a reference point in future studies. (4) Our analysis reveals distinct molecular characteristics of *E. coli* strains from the phylogroups B1 (shiga vs nonshiga), B2 (ST131 vs non-ST131), and E (ST11). We identified potential biological particles (a prophage, s2-ST131) that can serve as biological weapon for ST131 *E. coli* in bacterial competition. Several syntenic gene clusters found significantly coincident with s1-ST11 and/or s2-ST131 appear important for the respective strains’ pathogenicity.

## Methods

All methodical details and the datasets used are described in this section. In addition, a supplementary methods file with scripting support is available as Additional file 4 with this article.

### Dataset

The assembled genome sequences and NCBI Refseq annotations for prokaryotic data were searched for at the NCBI website (10 June 2021) [157, 158]. A total of 23,547 assembled genome sequences were associated with *E. coli*, out of which 1624 were of “complete genome” assembly level. To ensure that all the 1624 *E. coli* genomes followed the same annotation pipeline, we have downloaded the DNA and the protein sequences as well as the GFF (genome feature format) annotation files for all these genomes.

In order to streamline our analysis, we further evaluated the 1624 complete genomes of *E. coli* to guarantee that the selected genomes are of (1) high quality and (2) limited redundancy. Two genomes (Assembly IDs: GCF_000184185.1 and GCF_002925525.1) were excluded due to the difference between the Refseq and Genbank sequences as identified by the column “*paired_asm_comp*” in the NCBI Refseq summary file. This gives a total of 1622 genomes that remained to be analyzed. To remove highly similar genome sequences from the analysis, which in turn reduces redundancy in our dataset, we used fastANI version 1.32 [53] with default parameters to calculate the pairwise average nucleotide identity (ANI) of all the 1622 genomes. If the pairwise ANI is greater than 99.99%, then the genome with larger size was kept and the smaller genome was excluded from analysis. This step left us with 1324 genomes. The details of all 1624 genomes are provided in Supplementary file 1 (Additional file 3).

### *Escherichia coli* sequence typing, serotyping, and phylotyping

Given the complete genome sequences of *E. coli*, we performed in silico sequence typing using the stand-alone version of MLST version 2.0.4 [159] based on the Achtman criteria [160]. Seven housekeeping genes are profiled for MLST typing (i.e., *adk*, *fumC*, *gyrB*, *icd*, *mdh*, *purA*, and *recA*). The MLST database version 2.0.0 was downloaded on 2 August 2021. Eighteen sequences that have ambiguous sequence type or are unable to be typed were labelled “ST-unknown”.

For in silico serotyping, we used the stand-alone version of SerotypeFinder [161] version 2.0.1 together with the database version 1.0.0. SerotypeFinder is applied for O and H typing of bacterial sequences. O typing is based on genes *wzx*, *wzy*, *wzm*, and *wzt*. For H typing, the flagellin genes (i.e., *fliC*, *flkA*, *fllA*, *flmA*, and *flnA*) are analyzed. If there is no hit for O or H typing, we assigned the strain as “O-” or “H-”, respectively. If there is ambiguity, then we labelled the genomes as “O-unknown” or “H-unknown”, respectively.

*Escherichia coli* species can be divided into eight phylogroups, notably A, B1, B2, C, D, E, F, and G [51, 162]. We have used the software ClermonTyping [51] version 1.4.0 to perform in silico phylotyping of the *E. coli* genome sequences. ClermonTyping was performed on 11 August 2021. Phylogroups were assigned according to the output file phylogroups.txt from the software ClermonTyping.

### Prediction of *E. coli* antibiogram from the DNA sequences

We used ResFinder [163] v4.1 (27 May 2021) and ResFinder database (16 August 2021) to obtain the in silico antibiogram of the *E. coli* genomes. We ran the analysis using blastn for alignment. The gene matching was based on default parameters (i.e., sequence coverage of 60% and sequence identity of 80%). ResFinder associates 9 classes of antibiotics with 24 potential antimicrobial-resistant (AMR) targets (i.e., for aminoglycoside (3), beta-lactam (11), fluoroquinolone (2), folate pathway antagonist (2), fosfomycin (1), macrolide (1), phenicol (1), polymyxin (1), and tetracycline (2)). The antibiogram is provided as a matrix with genomes in rows and the 24 AMR targets in columns. The entries will be 0 for absence of the AMR target and 1 for its presence, respectively. For presence, we require the mapping to be with 100% sequence identity and 100% sequence coverage as we have noticed that there is little difference comparing to 60% sequence coverage and 80% sequence identity (see Supplementary file S6 in the zip package Additional file 3).

### Mapping of virulence factors in *E. coli* genome and assignment of pathogenicity likelihood

We used virulencefinder v2.0.3 (21 May 2020) [164] and its virulence database (from 29 May 2020) to obtain the in silico virulence factor mapping of the *E. coli* genomes. All parameters are the same as the in silico antibiogram mapping. There are a total of 177 virulence factors for *E. coli* available in the database. We calculated the virulence factor presence/absence matrix (PAM) with genomes in rows and the 177 virulence factors in columns. Similarly, the entries are 0 for absence and 1 for presence, respectively (see Supplementary file S7 in the zip package Additional file 3).

Given the virulence factor PAM, the number of virulence factors presence (VF count) for each genome is calculated and the quantile distribution is determined. The VF count ranges from 0 to 37 with the 25%, 50%, and 75% quantile threshold as 6, 14, and 22. Regarding their pathogenicity, *E. coli* strains can be classified into four categories (i.e., likely nonpathogenic (VF count < 6), likely virulent (VF count ranges from 6 to 14), highly virulent (VF count ranges from 14 to 22), and extremely virulent (VF count ≥ 22)).

### Finding clusters of homologous genes or gene families

Two publicly available software suites are used to investigate clusters of homologous genes or gene families (GF): CD-HIT [165] and ProteinOrtho [166]. We evaluated the clusters of GFs across different sequence identity (SeqID) [range 40 to 80%] and sequence length coverage (SeqLC) [range 50 to 90%]; subsequently, we compared the GFs identified from both CD-HIT and ProteinOrtho. The similarity between the GFs from both CD-HIT and ProteinOrtho is evaluated using Jaccard similarity index, which is defined as the ratio of common GFs between both methods divided by the union of GFs across both methods. Higher Jaccard similarity index (Eq. 1) indicates higher concordance between the two methods. Based on the Jaccard similarity index, we identify the optimal parameters (i.e., SeqID and SeqLC) to find GFs.

1 $$Jaccard\ \left(A,B\right)=\frac{\mid A\cap B\mid }{\mid A\cup B\mid}\bullet 100\%$$

### Pangenome development

Figure 2 shows the sequential steps in the pangenome development in accordance with our previously validated protocol [49]. Briefly, each protein sequence is tagged with its corresponding genome ID. Subsequently to streamline our analysis, three steps of filtering are used to significantly reduce the total number of 6,201,720 protein sequences from all genomes:

1. Proteins within a given genome are clustered at 98% SeqID with CD-HIT; the longest sequence is selected as representative (6,169,700 in total).
2. Clustering the set of representative sequences from step (1) with iterative application of CD-HIT with decreasing SeqID from 100 to 98% in 0.5% steps (essentially, this is a computation of the 98% SeqID core genome) results in 188 GFs with 248,921 sequence members.
3. The remaining 5,920,779 sequences from across all genomes are clustered with CD-HIT at 90% SeqID and 90% SeqLC. The longest sequences from each of the 52,798 clusters (including singletons) are extracted as cluster representatives.
4. Then, this reduced sequence set is processed with either CD-HIT or ProteinOrtho with given SeqID [range 40 to 80%] and SeqLC [range 50 to 90%] thresholds.

The resulting GFs from this step are then re-inflated with the protein sequences from clusters generated during sequence reduction steps (1) and (3), if they contain a sequence from the respective GF. The clusters from step (2) are added to set of clusters. Finally, if sequences of two resulting GFs contain the same PFAM domain, the two GFs are merged into one if their sequences can be clustered with CD-HIT or ProteinOrtho, respectively, using SeqID 40% and SeqLC 50%. This step leads to final set of gene families (GF) that will form the pangenome. The latter is represented as a presence/absence matrix (PAM) of size *n × m* where *n* is the number of GFs and *m* is the index of genomes. The entry for each cell in the matrix is either 1 or 0, which corresponds to presence or absence of the gene families in the genome, respectively.

### Correlating sequence type and phylogroup to the accessory genome presence/absence matrix (PAM)

To evaluate whether the sequence types or phylogroups of the *E. coli* genomes are correlated with the PAM, we focused on the sequence types with at least 10 genomes. There are only 21 sequence types with at least 10 genomes, which corresponds to 674 genomes altogether. The distribution of the sequence types and phylogroups of these 674 genomes are shown in Additional file 1: Fig. S1. The pangenome matrix based on ProteinOrtho method was filtered to include only these 674 genomes and their related GFs. This is to ensure that we include only the informative gene families for the correlation analysis. We found the resulting PAM matrix (sized 6244 × 674) to include only 6244 GFs with at least 10 genomes and at most 640 genomes, respectively. A heatmap profile of the PAM matrix is generated by applying unsupervised hierarchical clustering for both genomes and gene families accordingly (using Euclidean distance as distance metric and an agglomerative strategy).

### Identifying synteny clusters in *E. coli* subtype-specific gene family clusters

Among the clusters of GFs identified from the *E. coli* PAM of the accessories genome (i.e., 6244 × 674 PAM as described previously) that are clearly distinctive between strain groups, we found some that consist of genes closely localized in the respective genomes in the cases of ST11 and ST131. In order to identify synteny clusters, we performed several steps, i.e., (1) we re-annotate the NCBI GFF files for each ST131 and ST11 genomes based upon the cluster of homologous genes ID; (2) we filtered the modified annotation file to include only the specific GF clusters; (3) we walked through the filtered annotation file to identify operon or synteny cluster where the distance from one gene to another gene is at most 200 bp and there are at least 10 genes on the same strand; (4) we compared this synteny cluster across all the genomes in ST131 or ST11, respectively; and, finally, (5) we evaluated how common this cluster is in the specific *E. coli* ST131 or ST11 genomes. For step (3), if there is a skip gene in between two adjacent regions due to its common presence in all other *E. coli* genomes, then the synteny cluster will be extended to include this gene.

In order to avoid any potential bias in the analysis, we evaluated the ST131 or ST11 genomes according to the country, host species, and isolation source (collected from the NCBI Biosample annotation). We further evaluated the DNA length and GC content distribution. This is important to ensure that any observed conserved synteny cluster is not due to bias or contamination from the same source. We find that the genomes come from multiple sources with variable DNA length and GC content. This suggests that there is no confounding bias in the genomes analyzed.

### DNA and protein sequence analysis of synteny clusters

The DNA and protein sequences of the synteny clusters identified in specific *E. coli* subtypes (i.e., ST131 and ST11) were investigated with in-depth sequence analysis methods. The DNA sequences were evaluated at two levels, i.e., (1) how specific it is in the *E. coli* subtype of interest; and (2) whether there are hits to the NCBI non-redundant database excluding *E. coli* genomes. Briefly, the extracted DNA sequence of the synteny cluster from ST131 *E. coli* was searched against the ST131 and non-ST131 genome collections (with blastn v2.11.0+), respectively. Subsequently, the percentage of mapping hits are compared between the ST131 and non-ST131 genomes. This will give the specificity of the DNA sequences in our collection of genomes. On the other hand, the same DNA sequence is searched against the NCBI non-redundant database excluding *E. coli* genomes with blastn using default parameters. Similarly, the analysis is performed on ST11 specific synteny cluster.

The annotated protein sequences of the synteny clusters are investigated using (1) homology detection using HHPRED [167, 168] and (2) blastp (v2.11.0+) [169]. We applied the (3) in-house software suite ANNOTATOR [170, 171] to gain a quick overview of the proteins’ sequence domain architecture (globular domain functions and non-globular segments) and the potentially amino acid sequence-encoded biological functions.

### Finding prophages in *E. coli* genome

We use PHASTER [80, 172] to search for prophage genomes in the *E. coli* genomes. PHASTER categorizes the identified prophage into three categories, i.e., intact, questionable, and incomplete. It has been suggested that “questionable” and “incomplete” predicted prophages are often lacking some of the essential phage functions. Therefore, in this analysis, we will focus on the “intact” prophage signatures identified in the genomes.

### Analysis of GF associations in the accessory genome

CoinFinder v1.1 [114] was used to detect statistically significant associations and dissociations of GFs in the accessory genome of well-represented phylogenetic groups of *E. coli* strains. The program was applied to the set of 674 genomes from the most commonly observed *E. coli* sequence types as described above. We generated their phylogenetic tree based on the seven housekeeping genes used for MLST typing by following procedure:

(i) The nucleotide sequences of the seven housekeeping genes for each of the 674 genomes were concatenated.
(ii) The multiple sequence alignment (MSA) of the 674 concatenated sequences created with MUSCLE [173].
(iii) We identified the SNPs from the MSA file using the program SNP sites [174].
(iv) Finally, the phylogenetic tree was constructed with the help of raxML v8.2.11 [175].

The pangenome matrix was reformatted to suit the input needs for CoinFinder. We used the default threshold for filtering of gene families and applied the ad hoc *P*-value ≤ 1 × 10^−20^ as the threshold for picking up significant association.

### PFAM domain and COG annotation

HMMER3 v3.1b2 [176] is used to find known protein domains based on the PFAM release 33.1 [177]. For each of the protein sequences, the PFAM HMM profile is queried against the target sequences with E-value threshold of 0.001. The domain hits are compared across the different protein sequences for coherence.

For COG domain occurrence analysis, we annotate the softcore genome clusters/GFs (≥95% of all the genomes in this study, totally 3056 GFs). For each of the clusters, we extracted the protein fasta sequences. Subsequently, we performed multiple sequence alignment (MSA) of each cluster using MUSCLE [173]. Then, a Hidden Markov model (HMM) is built from each of the clusters’ MSA using *hmmbuild* from HMMER3 v3.1b2. The softcore HMM profile is queried against the COG database [61] (downloaded on 7 January 2021). The significant COG hits (with at least E-value of less than 0.001) were assigned to the softcore genome cluster accordingly. The functional code of the COG category is assigned based on the “cog-20.def.tab” from the COG database. COGs with multiple functional categories were assigned their first functional code assuming it as its most important functional category. Softcore genome clusters with no HMMER3 hits were labelled as “Unknown.” In addition, we also extracted the COGs from the COG database that are annotated as belonging to *E. coli* to serve as comparison and control. There are two *E. coli* strains being represented in the COG database, i.e., *E. coli* O157:H7 str. Sakai and *E. coli* str. K-12 substr. MG1655.

There are 20 functional codes available in the COG database following the general categorization by Satti et al. [47]. Briefly, functional codes D, M, N, O, T, U, V, W, and Y are categorized as “cellular processes and signaling,” functional codes A, B, J, K, and L are summarized as “information storage and processing,” functional codes C, E, F, G, H, I, P, and Q are grouped as “metabolism,” functional code X is categorized as “mobilome,” and functional codes R and S are seen as “poorly characterized”.

## Supplementary Information

**Additional file 1: Figures S1-S17.** This Additional file 1 provides 17 supplementary figures supporting the conclusions in the main text. **Fig. S1.** Genome distribution among the most common sequence types and phylogroups. Only common sequence types and phylogroups with at least 10 *E. coli* genomes are shown. The distribution of sequence types (A) and phylogroups (B) for the selected 674 *E. coli* genomes is illustrated. **Fig. S2.** Genome size and proteome size distribution among *E. coli* genomes. Boxplot illustration of the distribution of genome size (A) and proteome size (B) across different phylogroups of *E. coli* genomes. The OTH phylogroup represents 4 genomes in clade I (1), E or clade I (2) and unknown (1). **Fig.** S3. Genome distribution among sequence types and phylogroups. Barplot illustration of the distribution of sequence type (A) and phylogroups (B) among the 1,324 *E. coli* genomes. The y-axis represents the number of genomes. The horizontal line in (A) represents the threshold at number of genomes equal to 10. **Fig. S4.** The distribution of virulence categories across the 21 most common sequence types of *E. coli.* The distribution of virulence categories across the 21 common sequence types of *E. coli* ordered according to its phylogroups. Based on the total number of virulence factors (VFs) present in the genome, we categorized the genome into four virulence categories, i.e. (1) likely nonpathogenic (#VFs <6); (2) likely virulence (6 <= #VFs <14); (3) high virulence (14 <= #VFs < 22) and (4) very high virulence (#VFs >= 22). The phylogroup B1* represents phylogroup B1 with shiga toxin. **Fig. S5.** Distribution of COG categories in GFs of the reference genome and in the softcore genome. The distribution of COG categories for the gene families in (A) *E. coli* reference in the COG database; and (B) the softcore genome. **Fig. S6.** Distribution of COG categories in GFs in ST 131. The distribution of COG categories for the gene families that are (A) common in *E. coli* ST131; and (B) rare in *E. coli* ST131. **Fig. S7.** The presence of s1-ST131or s2-ST131 in ST131 and other *E. coli* genomes. The distribution of BLASTN coverage for (A) s1-ST131 and (B) s2-ST131 clusters in ST131 genomes; and non-ST131 genomes. The presence of s1-ST131or s2-ST131 cluster is shown by BLASTN coverage of 95% -100% whereas the absence of these clusters are shown in the BLASTN coverage 0% - 5%. The partial presence of these clusters is shown in between 5% to 95%. **Fig. S8.** Sequences similar to s1-ST131 among non-*E. coli* genomes. The top 20 hits of NCBI BLASTN to the nr-database excluding *E. coli* genomes for the s1-ST131 cluster. **Fig. S9.** Sequences similar to s2-ST131 among non-*E. coli* genomes. The top 20 hits of NCBI BLASTN to the nr-database excluding *E. coli* genomes for the s2-ST131 cluster. **Fig. S10.** Genome browser results of the s2-ST131 cluster. Genome browser results of the s2-ST131 cluster based upon GCF_000931565.1 as the representative *E. coli* ST131. The shown region is on chromosome NZ_CP010876.1 (2,042,977 – 2,066,794). The highlighted regions represent the nanomachine (tailocin), the capsid and the lysis-related genes. **Fig. S11.** The presence of s1-ST11or s2-ST11 in ST11 and other *E. coli* genomes. The distribution of BLASTN coverage for (A) s1-ST11 and (B) s2-ST11 clusters in the ST11 genomes; and non-ST11 genomes. The presence of s1-ST11 or s2-ST11 cluster is shown by BLASTN coverage of 95% -100% whereas the absence of these clusters are shown in the BLASTN coverage 0% - 5%. The partial presence of these clusters is shown in between 5% to 95%. **Fig. S12.** Sequences similar to s1-ST11 among non-*E. coli* genomes. The top 20 hits of NCBI BLASTN to the nr-database excluding *E. coli* genomes for the s1-ST11 cluster. **Fig. S13.** Sequences similar to s2-ST11 among non-*E. coli* genomes. The top 20 hits of NCBI BLASTN to the nr-database excluding *E. coli* genomes for the s2-ST11 cluster. **Fig. S14.** Analyzing s1-ST11 with antiSMASH. antiSMASH result from analyzing s1-ST11. It is observed that the aryl polyene biosynthetic gene is observed in the cluster. Also, there are other additional biosynthetic genes as shown in the genes’ cluster. **Fig. S15.** Analyzing s2-ST11 with antiSMASH. antiSMASH result from analyzing s2-ST11. It is observed that the aryl polyene biosynthetic gene cluster is observed with 94% similarity. BGC0000836 is the biosynthetic cluster in the UPEC strain CFT073. **Fig. S16.** Histogram of the -log10 *P*-value generated with CoinFinder. Histogram of the -log10 *P*-value of the pairwise GF association generated from the CoinFinder output for the all pairwise comparisons. The figure on the right is the enlarged section of the distribution with the y-axis truncated at 10^6^. The *P*-value 1 x 10^-20^ is selected as the *ad hoc* cut-off criterion for significant pairwise comparisons in this study. **Fig. S17.** Distribution of significantly associated GFs (associated GF cluster sizes). The distribution of number of associated GFs for each significant GF (*P*-value <= 1 x 10^-20^). The number of associated GFs for each significant GF ranges from 1 to 607. Though there are overwhelmingly high number of GFs with fewer than 50 associated GFs, there are quite a substantial number of GFs with many associated GFs as well, especially those with more than 300 associated GFs.

**Additional file 2: Tables S1-S10.** This Additional file 2 provides 10 supplementary tables supporting the conclusions in the main text. **Tab. S1.** Performance evaluation of the pangenome development. We list the numbers of clusters of homologous genes/proteins across different range of SeqID and SeqLC. **Tab. S2.** Effect of SeqID and SeqLC on the number of clusters. To evaluate the effect of SeqLC, we evaluate the number of clusters across different seqID at each SeqLC threshold using linear regression. The slope represents the amount of change with respect to every increase in SeqLC. Similarly, to evaluate the effect of SeqID, the number of clusters across different SeqLC threshold is evaluated and the slope is calculated. The evaluation is done on both methods, *i.e.*, CD-HIT and ProteinOrtho. **Tab. S3.** Synteny clusters among the common genes specific to ST131 *Escherichia coli*. **Tab. S4.** Annotating the s1-ST131 cluster. *Pseudomonas aeruginosa* genes are represented with prefix PA. **Tab. S5.** Annotating the s2-ST131 cluster. *Pseudomonas aeruginosa* genes are represented with prefix PA. **Tab. S6.** Synteny clusters among the common genes specific to ST11 *Escherichia coli*. **Tab. S7.** The number of gene families (GFs) associated to s2-ST131 gene families. The identification of significantly associated gene families was carried out using CoinFinder based on a *p*-value <= 1^-20^ cutoff. **Tab. S8.** Supplementary Table S8: The number of gene families (GFs) associated to s1-ST11 gene families. The identification of significantly associated gene families was carried out using CoinFinder based on a *p*-value <= 1^-20^ cutoff. **Tab. S9.** Synteny cluster analysis of the GFs associated with s2-ST131. The inter-gene distance is kept at the maximum 1000 bp with at least 10 members per cluster. The s1-ST131 is excluded in this table. **Tab. S10.** Supplementary Table S10: Synteny cluster analysis of the GFs associated with s1-ST11. The inter-gene distance is kept at the maximum of 1000 bp with at least 10 members per cluster. The s2-ST11 cluster is excluded in this table.

**Additional file 3. **The Additional file 3 is a compressed file library (zip package) containing 11 files. **File 1** genome list with serotypes, sequence types, phylogroups, etc. **File 2A** pangenome matrix determined with CD-HIT. **File 2B** softcore genome GF list determined with CD-HIT. **File 3A** pangenome matrix determined with ProteinOrtho. **File 3B** softcore genome GF list determined with ProteinOrtho. **File 4** the GFs of the six distinctive clusters. **File 5** summary of the strains’ virulence and antibiotics resistance. **File 6** antibiogram data. **File 7** virulence factor PAM. **File 8** coincident pairwise association results from CoinFinder. README.

**Additional file 4: Supplementary Methods.** The Supplementary Methods file is available both at GitHub https://github.com/biierwint/ecoli_pangenome [180] as well as Additional file 4 with this article.

## Data Availability

All data generated and analyzed during this study are included in this published article and its supplementary information files. Additional file 1 provides 17 supplementary figures. Additional file 2 contains 10 supplementary tables. The zip package Additional file 3 provides a README with content description of ten further files contained in the package: genome list (File 1), pangenome matrix (File 2A and File 3A) and softcore genome GF list (File 2B and File 3B) determined with CD-HIT and ProteinOrtho, respectively, the GFs of the six distinctive clusters (File 4), virulence and antibiogram data (Files 5, 6 and 7), and coincident pairwise association results derived with CoinFinder (File 8). Further additional materials are available for download at the GitHub repository: https://github.com/biierwint/ecoli_pangenome [178]. The entry includes the details of protein IDs for each gene family (subfolder: pangenome-results) both for the pangenome and the softcore genome as well as the collection of protein sequences used in this study (subfolder: protein-sequences). The Supplementary Methods file is available both at GitHub as well as Additional file 4 with this article.

## Abbreviations

AIEC: Adherent invasive *E. coli*
AMR: Anti-microbial resistance
APEC: Avian pathogenic *E. coli*
COG: Cluster of orthologous genes
DAEC: Diffusely adherent *E. coli*
DNA: Deoxyribonucleic acid
*E. coli*: *Escherichia coli*
EAEC: Enteroaggregative *E. coli*
EHEC: Enterohemorrhagic *E. coli*
EPEC: Enteropathogenic *E. coli*
ETEC: Enterotoxigenic *E. coli*
ExPEC: Extraintestinal pathogenic *E. coli*
GF: Gene/protein family (family of sequentially similar genes/proteins thought to be orthologous)
HMM: Hidden Markov model
InPEC: Intestinal pathogenic *E. coli*
NCBI: National Center of Biological Information USA
NMEC: Neonatal meningitis-associated *E. coli*
PAM: Presence/absence matrix (with indices for gene families and genomes in the pangenome)
SeqID: Sequence identity
SeqLC: Sequence length coverage
ST: Sequence type
UPEC: Urinary pathogenic *E. coli*
UTI: Urinary tract infection
VF: Virulence factor
