# Supplementary material for: To kill or to be killed: pangenome analysis of Escherichia coli strains reveals a tailocin specific for pandemic ST131
Source: BMC Biol. 2022 Jun 16;20:146. doi: 10.1186/s12915-022-01347-7 (PMC9205054; doi:10.1186/s12915-022-01347-7)
Supplement: Supplementary file 1 — Additional file 1: Figures S1-S17. This Additional file 1 provides 17 supplementary figures supporting the conclusions in the main text. Fig. S1. Genome distribution among the most common sequence types and phylogroups. Only common sequence types and phylogroups with at least 10 E. coli genomes are shown. The distribution of sequence types (A) and phylogroups (B) for the selected 674 E. coli genomes is illustrated. Fig. S2. Genome size and proteome size distribution among E. coli genomes. Boxplot illustration of the distribution of genome size (A) and proteome size (B) across different phylogroups of E. coli genomes. The OTH phylogroup represents 4 genomes in clade I (1), E or clade I (2) and unknown (1). Fig. S3. Genome distribution among sequence types and phylogroups. Barplot illustration of the distribution of sequence type (A) and phylogroups (B) among the 1,324 E. coli genomes. The y-axis represents the number of genomes. The horizontal line in (A) represents the threshold at number of genomes equal to 10. Fig. S4. The distribution of virulence categories across the 21 most common sequence types of E. coli. The distribution of virulence categories across the 21 common sequence types of E. coli ordered according to its phylogroups. Based on the total number of virulence factors (VFs) present in the genome, we categorized the genome into four virulence categories, i.e. (1) likely nonpathogenic (#VFs <6); (2) likely virulence (6 <= #VFs <14); (3) high virulence (14 <= #VFs < 22) and (4) very high virulence (#VFs >= 22). The phylogroup B1* represents phylogroup B1 with shiga toxin. Fig. S5. Distribution of COG categories in GFs of the reference genome and in the softcore genome. The distribution of COG categories for the gene families in (A) E. coli reference in the COG database; and (B) the softcore genome. Fig. S6. Distribution of COG categories in GFs in ST 131. The distribution of COG categories for the gene families that are (A) common in E. coli ST131; and ( [file 12915_2022_1347_MOESM1_ESM.pdf]

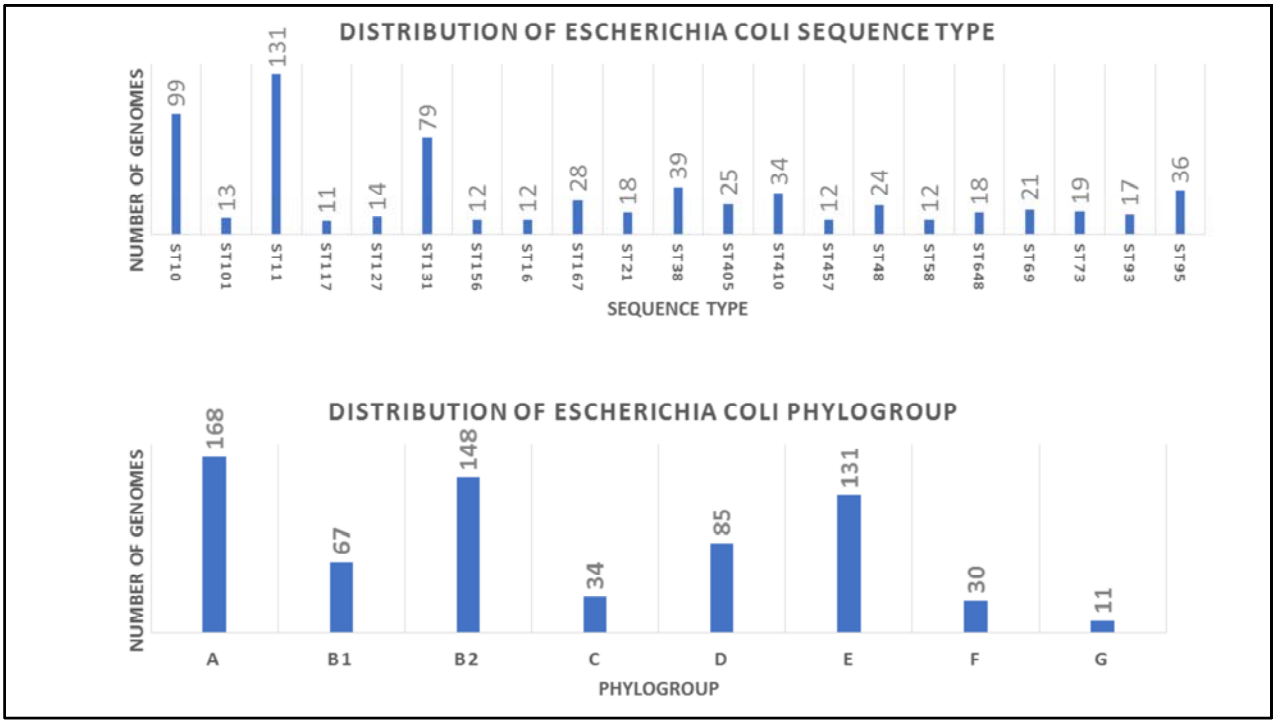

Supplementary Figure S1: Genome distribution among the most common sequence types and phylogroups

Only common sequence types and phylogroups with at least 10 *E. coli* genomes are shown. The distribution of sequence types (A) and phylogroups (B) for the selected 674 *E. coli* genomes is illustrated.

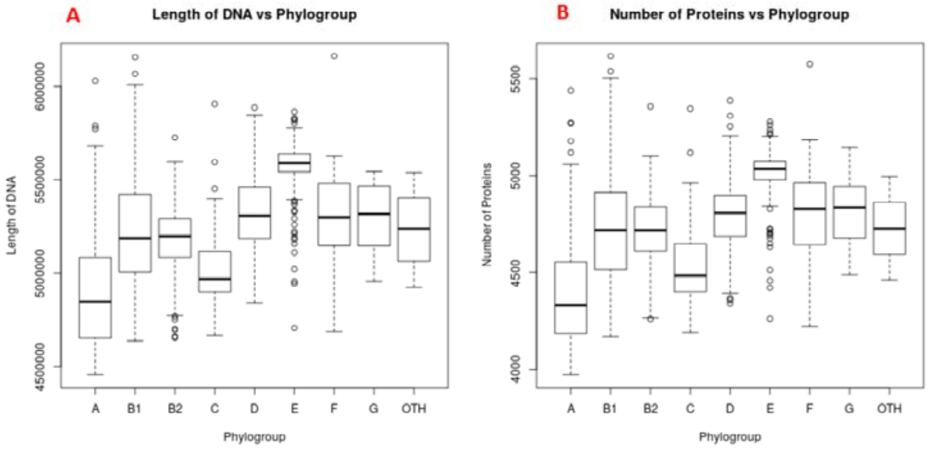

Supplementary Figure S2: Genome size and proteome size distribution among *E. coli* genomes

Boxplot illustration of the distribution of genome size (A) and proteome size (B) across different phylogroups of *E. coli* genomes. The OTH phylogroup represents 4 genomes in clade I (1), E or clade I (2) and unknown (1).

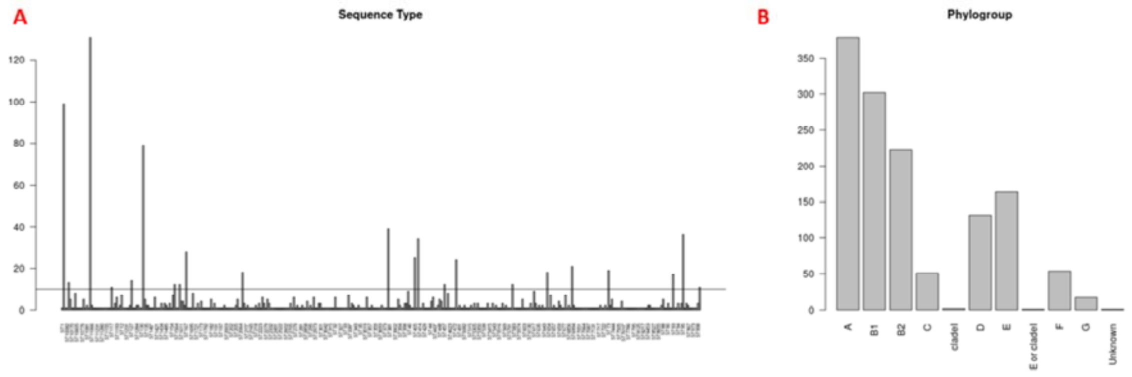

Supplementary Figure S3: Genome distribution among sequence types and phylogroups
Barplot illustration of the distribution of sequence type (A) and phylogroups (B) among the 1,324 *E. coli* genomes. The y-axis represents the number of genomes. The horizontal line in (A) represents the threshold at number of genomes equal to 10.

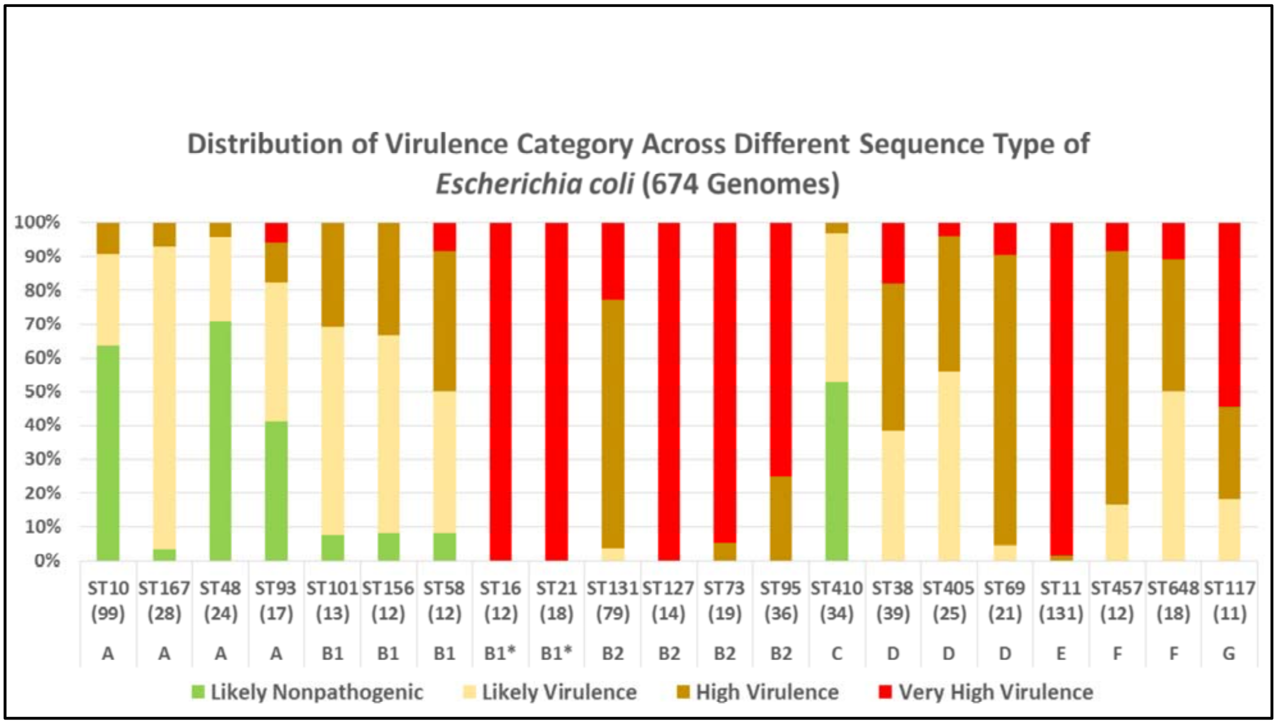

Supplementary Figure S4: The distribution of virulence categories across the 21 most common sequence types of *E. coli*

The distribution of virulence categories across the 21 common sequence types of *E. coli* ordered according to its phylogroups. Based on the total number of virulence factors (VFs) present in the genome, we categorized the genome into four virulence categories, i.e. (1) likely nonpathogenic (#VFs <6); (2) likely virulence (6 <= #VFs <14); (3) high virulence (14 <= #VFs < 22) and (4) very high virulence (#VFs >= 22). The phylogroup B1* represents phylogroup B1 with shiga toxin.

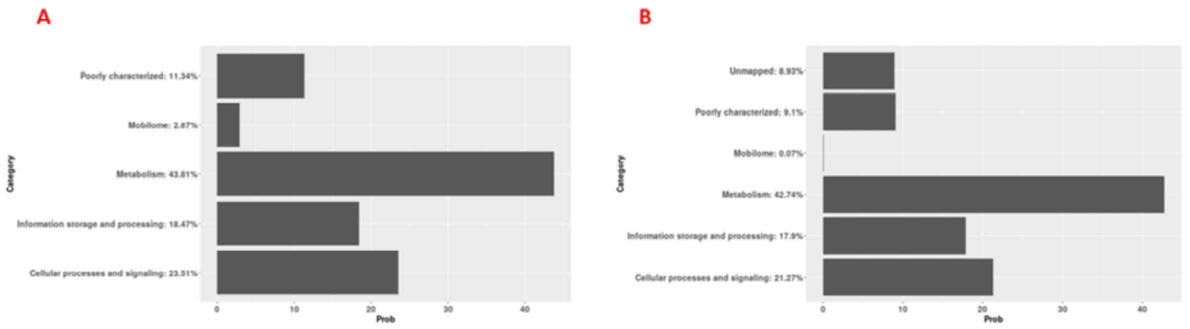

Supplementary Figure S5: Distribution of COG categories in GFs of the reference genome and in the softcore genome

The distribution of COG categories for the gene families in (A) *E. coli* reference in the COG database; and (B) the softcore genome.

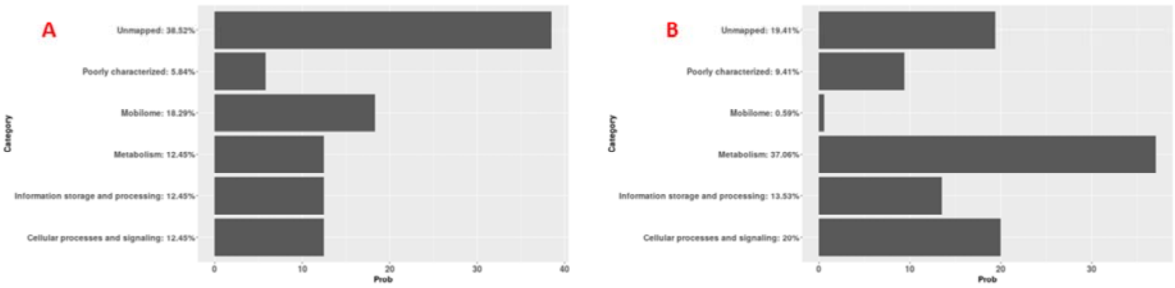

Supplementary Figure S6: Distribution of COG categories in GFs in ST 131

The distribution of COG categories for the gene families that are (A) common in *E. coli* ST131; and (B) rare in *E. coli* ST131.

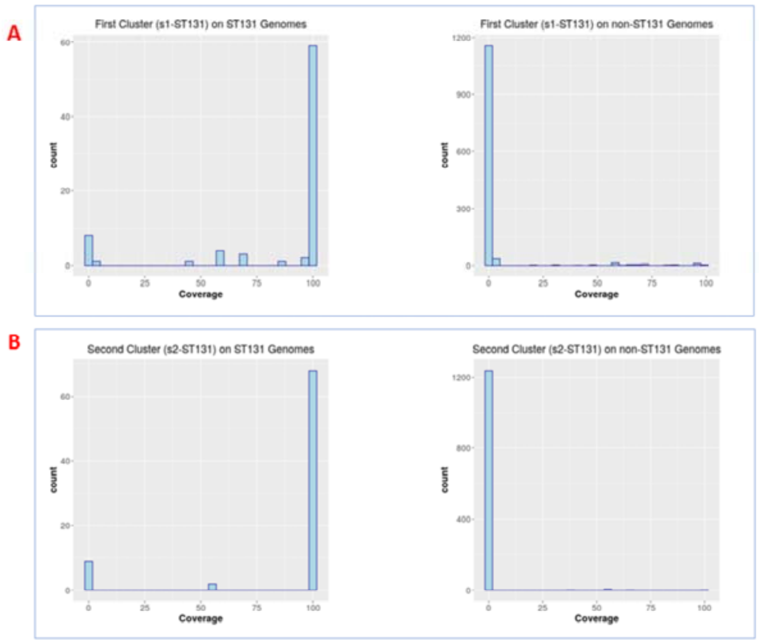

Supplementary Figure S7: The presence of s1-ST131or s2-ST131 in ST131 and other *E. coli* genomes

The distribution of BLASTN coverage for (A) s1-ST131 and (B) s2-ST131 clusters in ST131 genomes; and non-ST131 genomes. The presence of s1-ST131or s2-ST131 cluster is shown by BLASTN coverage of 95% -100% whereas the absence of these clusters are shown in the BLASTN coverage 0% - 5%. The partial presence of these clusters is shown in between 5% to 95%.

	Description	Scientific Name	Max Score	Total Score	Query Cover	E value	Per. Ident	Acc. Len	Accession
☑	Klebsiella michiganensis THO-011 DNA, complete genome	Klebsiella mich...	42435	44373	100%	0.0	100.00%	5935402	AP022547.1
☑	Klebsiella pneumoniae isolate INF247-sc-2280148 genome assembly, chromosome: 1	Klebsiella pneu...	41899	44379	100%	0.0	99.88%	5206451	LR890562.1
☑	Klebsiella pneumoniae strain BA2105 chromosome, complete genome	Klebsiella pneu...	41894	65633	100%	0.0	99.87%	5420464	CP060421.1
☑	TPA: Myoviridae sp. isolate ctWAk2, partial genome	Myoviridae sp.	40996	42279	97%	0.0	99.76%	27303	BK037528.1
☑	TPA: Myoviridae sp. isolate ctYPI2, partial genome	Myoviridae sp.	34095	40392	96%	0.0	98.74%	38350	BK044152.1
☑	Klebsiella aerogenes strain G7 chromosome, complete genome	Klebsiella aero...	31157	38349	90%	0.0	98.34%	5452368	CP011539.1
☑	Enterobacter aerogenes EA1509E complete genome	Klebsiella aero...	31157	38625	90%	0.0	98.34%	5419609	FO203355.1
☑	Klebsiella pneumoniae strain B16KP0089 chromosome, complete genome	Klebsiella pneu...	29486	43473	100%	0.0	99.99%	5376541	CP052546.1
☑	Escherichia fergusonii strain RHB33-C04 chromosome, complete genome	Escherichia fer...	28179	42224	99%	0.0	98.52%	4729689	CP057215.1
☑	Escherichia fergusonii strain RHB33-C07 chromosome, complete genome	Escherichia fer...	28179	42224	99%	0.0	98.52%	4729644	CP057210.1
☑	Escherichia fergusonii strain RHB32-C05 chromosome, complete genome	Escherichia fer...	26424	42236	99%	0.0	98.89%	4697820	CP057243.1
☑	TPA: Siphoviridae sp. isolate cteTN1, partial genome	Siphoviridae sp.	26051	36089	88%	0.0	98.12%	36302	BK021514.1
☑	Salmonella enterica subsp. enterica serovar Typhi strain SGB90 genome assembly, chromosome: 1	Salmonella ent...	22631	31971	87%	0.0	96.48%	4784041	LT904870.2
☑	Salmonella enterica subsp. enterica serovar Typhi strain SGB89 genome assembly, chromosome: 1	Salmonella ent...	22631	31969	88%	0.0	96.48%	4777711	LT904882.1
☑	Salmonella enterica subsp. enterica serovar Typhi str. Ty2 strain 4316STDY6559672 genome assembly...	Salmonella ent...	22626	31971	87%	0.0	96.47%	4837118	LR590082.1
☑	Salmonella enterica subsp. enterica serovar Typhi str. Ty2 strain 4316STDY6559669 genome assembly...	Salmonella ent...	22626	31965	87%	0.0	96.47%	4839011	LR590081.1
☑	Salmonella enterica subsp. enterica serovar Typhi strain 343077_228140 chromosome, complete gen...	Salmonella ent...	22626	32064	88%	0.0	96.47%	4790593	CP029866.1
☑	Salmonella enterica subsp. enterica serovar Typhi strain 343077_213147 chromosome, complete gen...	Salmonella ent...	22626	32189	88%	0.0	96.47%	4897593	CP029897.1
☑	Salmonella enterica subsp. enterica serovar Typhi strain 343076_269157 chromosome, complete gen...	Salmonella ent...	22626	32035	87%	0.0	96.47%	4791108	CP029881.1
☑	Salmonella enterica subsp. enterica serovar Typhi strain 343076_253155 chromosome, complete gen...	Salmonella ent...	22626	32062	88%	0.0	96.47%	4791587	CP029890.1

Supplementary Figure S8: Sequences similar to s1-ST131 among non-*E. coli* genomes
The top 20 hits of NCBI BLASTN to the nr-database excluding *E. coli* genomes for the s1-ST131 cluster.

Description	Scientific Name	Max Score	Total Score	Query Cover	E value	Per. Ident	Acc. Len	Accession
TPA: Bacteriophage sp. isolate ctr7R7, partial genome	Bacteriophage sp.	43979	43979	100%	0.0	100.00%	43864	BK034715.1
TPA: Siphoviridae sp. isolate ctvuw3, partial genome	Siphoviridae sp.	19195	33849	95%	0.0	93.56%	46702	BK058252.1
TPA: Bacteriophage sp. isolate ct7ui22, partial genome	Bacteriophage sp.	18980	30421	90%	0.0	93.13%	45119	BK022544.1
Escherichia sp. SCLE84 chromosome, complete genome	Escherichia sp. SCLE84	18875	30791	90%	0.0	92.99%	5024521	CP051430.1
Escherichia albertii strain 1551-2 chromosome, complete genome	Escherichia albertii	18785	35349	94%	0.0	93.87%	4730877	CP025317.1
TPA: Bacteriophage sp. isolate ctTzY2, partial genome	Bacteriophage sp.	5254	9390	24%	0.0	96.26%	12739	BK021032.1
Shigella dysenteriae strain NCTC 9718 chromosome, complete genome	Shigella dysenteriae	1341	1450	4%	0.0	91.89%	4371869	CP026786.1
Shigella dysenteriae strain 80-547 chromosome, complete genome	Shigella dysenteriae	1341	1341	4%	0.0	91.89%	4391331	CP026784.1
Shigella dysenteriae strain 08-3380 chromosome, complete genome	Shigella dysenteriae	1341	1450	4%	0.0	91.89%	4464195	CP026782.1
Shigella dysenteriae strain 53-3937 chromosome, complete genome	Shigella dysenteriae	1341	1450	4%	0.0	91.89%	4382743	CP026780.1
Shigella dysenteriae strain 07-3308 chromosome, complete genome	Shigella dysenteriae	1341	1450	4%	0.0	91.89%	4382687	CP026878.1
Shigella dysenteriae strain 69-3818 chromosome, complete genome	Shigella dysenteriae	1341	1450	4%	0.0	91.89%	4390268	CP026777.1
Shigella dysenteriae strain BU53M1 chromosome, complete genome	Shigella dysenteriae	1341	1450	4%	0.0	91.89%	4409083	CP024466.1
Shigella dysenteriae 1617, complete genome	Shigella dysenteriae 1617	1341	1341	4%	0.0	91.89%	4302517	CP006736.1
Shigella dysenteriae Sd197, complete genome	Shigella dysenteriae Sd197	1341	1450	4%	0.0	91.89%	4369232	CP000034.1
Shigella dysenteriae strain ATCC 13313 chromosome, complete genome	Shigella dysenteriae	1336	1444	4%	0.0	91.79%	4395762	CP026774.1
Shigella dysenteriae strain HNCMB 20080 chromosome, complete genome	Shigella dysenteriae	1336	1444	4%	0.0	91.79%	4393662	CP061527.1
Salmonella enterica strain NCTC10436 genome assembly, chromosome: 1	Salmonella enterica	747	992	16%	0.0	73.08%	4787620	LS483428.1
Escherichia albertii strain 2014C-4015 chromosome, complete genome	Escherichia albertii	139	279	0%	1e-26	86.05%	4623903	CP034166.1
Escherichia albertii DNA, complete genome, strain: EC06-170	Escherichia albertii	137	137	0%	4e-26	85.94%	4657167	AP014857.1

Supplementary Figure S9: Sequences similar to s2-ST131 among non-*E. coli* genomes
The top 20 hits of NCBI BLASTN to the nr-database excluding *E. coli* genomes for the s2-ST131 cluster.

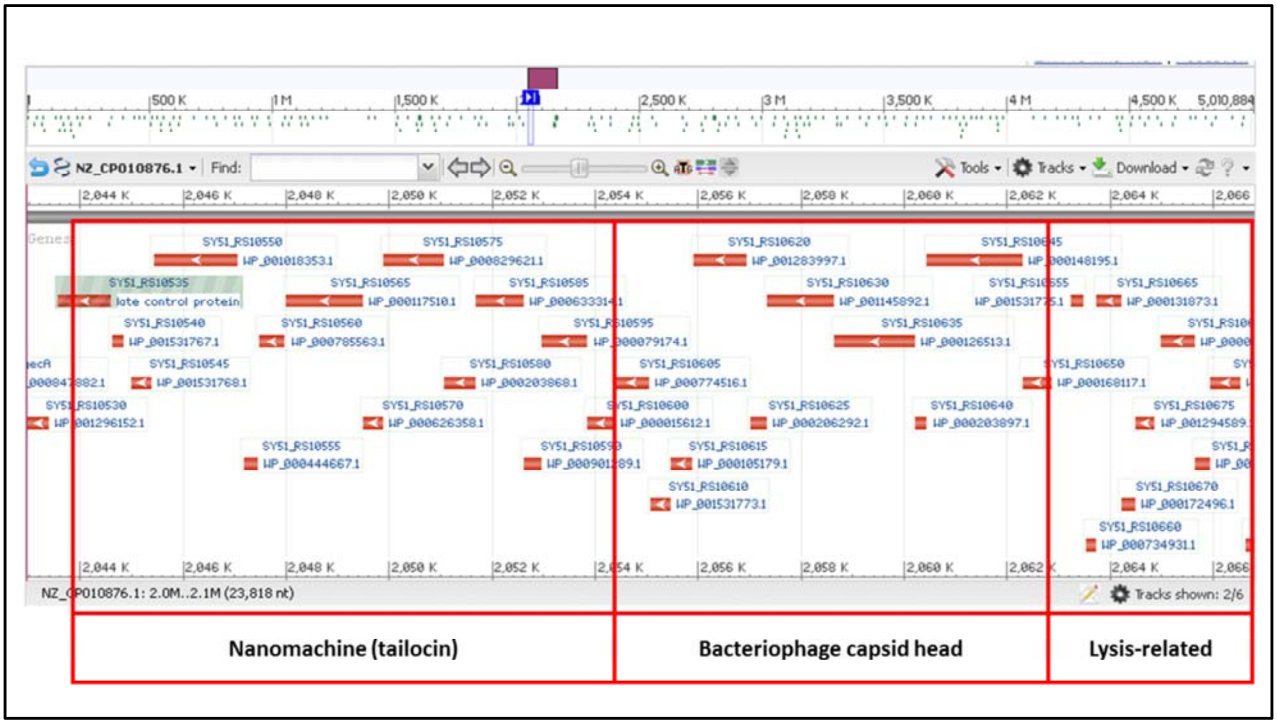

Supplementary Figure S10: Genome browser results of the s2-ST131 cluster
Genome browser results of the s2-ST131 cluster based upon GCF_000931565.1 as the representative *E. coli* ST131. The shown region is on chromosome NZ_CP010876.1 (2,042,977 – 2,066,794). The highlighted regions represent the nanomachine (tailocin), the capsid and the lysis-related genes.

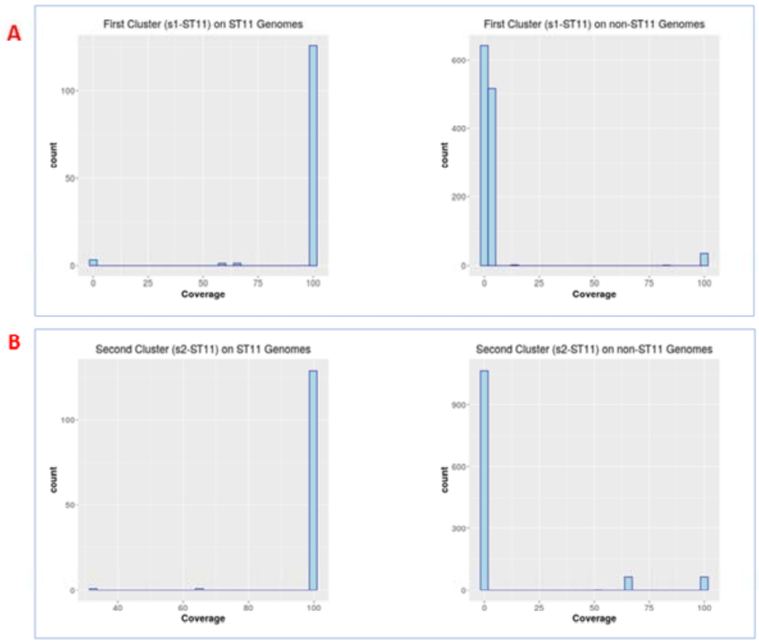

Supplementary Figure 11: The presence of s1-ST11or s2-ST11 in ST11 and other *E. coli* genomes

The distribution of BLASTN coverage for (A) s1-ST11 and (B) s2-ST11 clusters in the ST11 genomes; and non-ST11 genomes. The presence of s1-ST11 or s2-ST11 cluster is shown by BLASTN coverage of 95% -100% whereas the absence of these clusters are shown in the BLASTN coverage 0% - 5%. The partial presence of these clusters is shown in between 5% to 95%.

Description	Scientific Name	Max Score	Total Score	Query Cover	E value	Per. Ident	Acc. Len	Accession
Enterobacter mori strain CX01 chromosome, complete genome	Enterobacter mori	2276	4303	44%	0.0	76.32%	4966908	CP055276.1
Enterobacter mori strain BC01 chromosome, complete genome	Enterobacter mori	2259	4286	44%	0.0	76.25%	4818618	CP084692.1
Enterobacter cloacae strain D41-sc-1712200 chromosome, complete genome	Enterobacter cloacae	2122	4237	45%	0.0	75.70%	4826830	CP056779.1
Enterobacter cloacae strain DSM 26481 chromosome	Enterobacter cloacae	2122	4270	45%	0.0	75.70%	4896691	CP056117.1
Enterobacter hormaechei subsp. xiangfangensis strain UM_CRE-14 chromosome	Enterobacter hormaechei ...	2100	4215	41%	0.0	77.38%	4924340	CP023430.1
Enterobacter hormaechei subsp. xiangfangensis strain OSUVMCKPC4-2 chromosome, comple...	Enterobacter hormaechei ...	2100	4210	41%	0.0	77.35%	4752717	CP029246.1
Enterobacter hormaechei subsp. xiangfangensis strain OSUKPC4_L chromosome, complete g...	Enterobacter hormaechei ...	2100	4210	41%	0.0	77.35%	4753669	CP024908.1
Enterobacter xiangfangensis strain LMG27195, complete genome	Enterobacter hormaechei ...	2100	4215	41%	0.0	77.38%	4661849	CP017183.1
Enterobacter hormaechei subsp. xiangfangensis strain 34978 chromosome, complete genome	Enterobacter hormaechei ...	2100	4215	41%	0.0	77.38%	4930963	CP012165.1
Enterobacter sp. BWH 37 chromosome, complete genome	Enterobacter sp. BWH 37	2100	4210	41%	0.0	77.35%	4794271	CP072970.1
Enterobacter sp. MGH 7 chromosome, complete genome	Enterobacter sp. MGH 7	2100	4215	41%	0.0	77.38%	4880473	CP072945.1
Enterobacter cloacae complex sp. strain AR_0136 chromosome, complete genome	Enterobacter cloacae co...	2100	4215	41%	0.0	77.38%	4933551	CP021902.1
Enterobacter cloacae complex sp. strain AR_0053 chromosome, complete genome	Enterobacter cloacae co...	2100	4215	41%	0.0	77.38%	4974147	CP021776.1
Enterobacter cloacae complex sp. strain AR_0002 chromosome, complete genome	Enterobacter cloacae co...	2100	4215	41%	0.0	77.35%	4882039	CP018814.1
Enterobacter hormaechei subsp. xiangfangensis strain Ec61 chromosome, complete genome	Enterobacter hormaechei ...	2100	4210	41%	0.0	77.38%	4887670	CP053103.1
Enterobacter hormaechei subsp. xiangfangensis strain WCHEX045001 chromosome, complet...	Enterobacter hormaechei ...	2095	3568	34%	0.0	77.32%	4698270	CP043382.1
Enterobacter hormaechei strain 388 chromosome, complete genome	Enterobacter hormaechei	2095	4215	41%	0.0	77.35%	4656223	CP021167.1
Enterobacter hormaechei strain 234 chromosome, complete genome	Enterobacter hormaechei	2095	4215	41%	0.0	77.35%	4656282	CP021162.1
Enterobacter cloacae complex sp. ECL112 chromosome, complete genome	Enterobacter cloacae co...	2095	3568	34%	0.0	77.35%	4727601	CP077661.1
Enterobacter hormaechei strain CEent1 chromosome	Enterobacter hormaechei	2095	4215	41%	0.0	77.32%	4803493	CP058553.1

Supplementary Figure S12: Sequences similar to s1-ST11 among non-*E. coli* genomes
The top 20 hits of NCBI BLASTN to the nr-database excluding *E. coli* genomes for the s1-ST11 cluster.

Description	Scientific Name	Max Score	Total Score	Query Cover	E value	Per. Ident	Acc. Len	Accession
Escherichia sp. E4742 chromosome, complete genome	Escherichia sp. E4742	23965	23965	100%	0.0	95.47%	5120753	CP040443.1
Escherichia fergusonii strain RHB24-C06 chromosome, complete genome	Escherichia fergusonii	23115	23115	100%	0.0	94.45%	4457616	CP057505.1
Escherichia fergusonii strain RHB24-C08 chromosome, complete genome	Escherichia fergusonii	23115	23115	100%	0.0	94.45%	4457616	CP057504.1
Escherichia fergusonii strain FDAARGOS_1499 chromosome, complete genome	Escherichia fergusonii	22748	22748	100%	0.0	94.01%	4588714	CP083638.1
Escherichia fergusonii strain FDAARGOS_1032 chromosome, complete genome	Escherichia fergusonii	22748	22748	100%	0.0	94.01%	4590101	CP066085.1
Escherichia fergusonii ATCC 35469 chromosome, complete genome	Escherichia fergusonii ATCC 35469	22748	22748	100%	0.0	94.01%	4588711	CU928158.2
Escherichia fergusonii strain 6S41-1 chromosome, complete genome	Escherichia fergusonii	22524	22524	100%	0.0	93.74%	4724978	CP079884.1
Escherichia fergusonii strain RHB38-C07 chromosome, complete genome	Escherichia fergusonii	22524	22524	100%	0.0	93.74%	4730551	CP057093.1
Escherichia fergusonii strain RHB41-C15 chromosome, complete genome	Escherichia fergusonii	22524	22524	100%	0.0	93.74%	4690654	CP056968.1
Escherichia fergusonii strain RHB41-C20 chromosome, complete genome	Escherichia fergusonii	22524	22524	100%	0.0	93.74%	4690654	CP056961.1
Escherichia fergusonii strain 5zf15-2-1 chromosome, complete genome	Escherichia fergusonii	22513	22513	100%	0.0	93.73%	4934492	CP079891.1
Escherichia fergusonii strain ATCC 35473 chromosome, complete genome	Escherichia fergusonii	22502	22502	100%	0.0	93.72%	4657720	CP042942.1
Escherichia fergusonii strain FDAARGOS 1438 chromosome, complete genome	Escherichia fergusonii	22480	22480	100%	0.0	93.69%	4543156	CP077242.1
Escherichia fergusonii strain RHB17-C11 chromosome, complete genome	Escherichia fergusonii	22480	22480	100%	0.0	93.69%	4549644	CP057692.1
Escherichia fergusonii strain HNCF11W chromosome, complete genome	Escherichia fergusonii	22474	22474	100%	0.0	93.68%	4584794	CP053045.1
Escherichia fergusonii strain ATCC 35471 chromosome, complete genome	Escherichia fergusonii	22474	22474	100%	0.0	93.68%	4638019	CP042945.1
Escherichia fergusonii strain RHB28-C13 chromosome, complete genome	Escherichia fergusonii	22458	22458	100%	0.0	93.66%	4718083	CP055675.1
Escherichia fergusonii strain RHB02-C22 chromosome, complete genome	Escherichia fergusonii	22452	22452	100%	0.0	93.66%	4727820	CP058057.1
Escherichia fergusonii strain RHB02-C18 chromosome, complete genome	Escherichia fergusonii	22452	22452	100%	0.0	93.66%	4727976	CP055850.1
Escherichia fergusonii strain RHB02-C15 chromosome, complete genome	Escherichia fergusonii	22452	22452	100%	0.0	93.66%	4727977	CP055860.1

Supplementary Figure S13: Sequences similar to s2-ST11 among non-*E. coli* genomes
The top 20 hits of NCBI BLASTN to the nr-database excluding *E. coli* genomes for the s2-ST11 cluster.

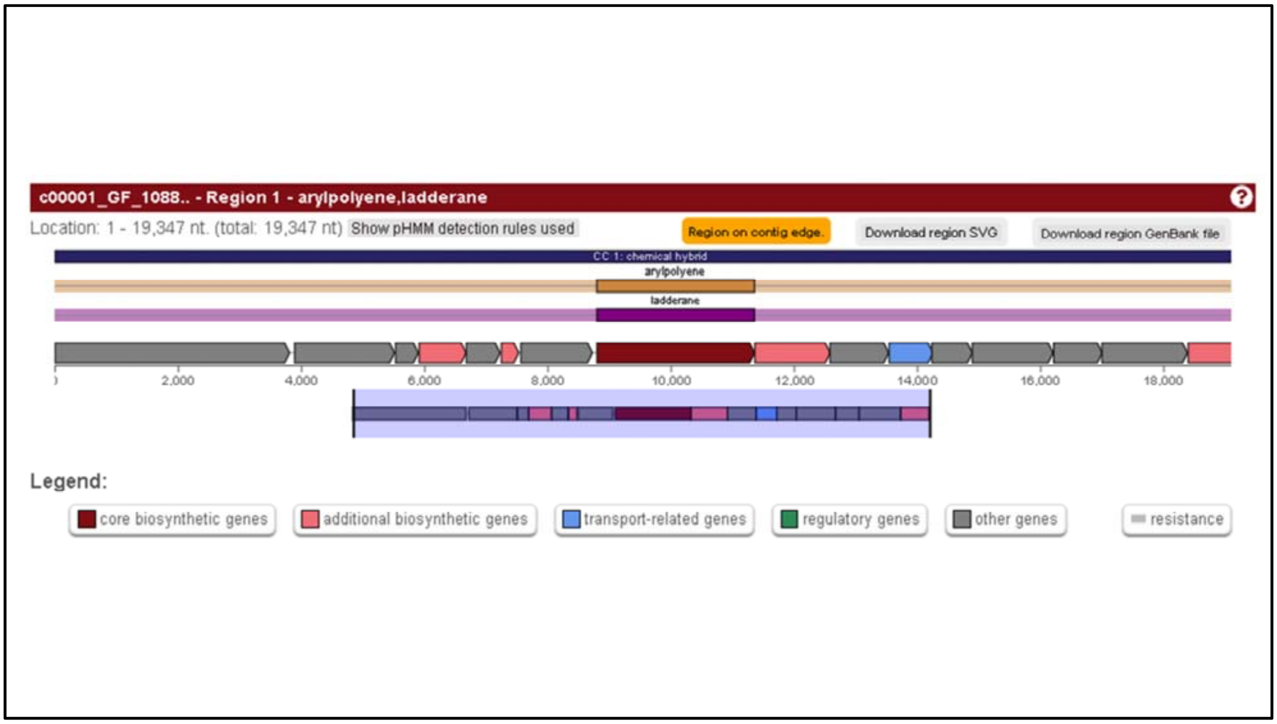

Supplementary Figure S14: Analyzing s1-ST11 with antiSMASH
antiSMASH result from analyzing s1-ST11. It is observed that the aryl polyene biosynthetic gene is observed in the cluster. Also, there are other additional biosynthetic genes as shown in the genes' cluster.

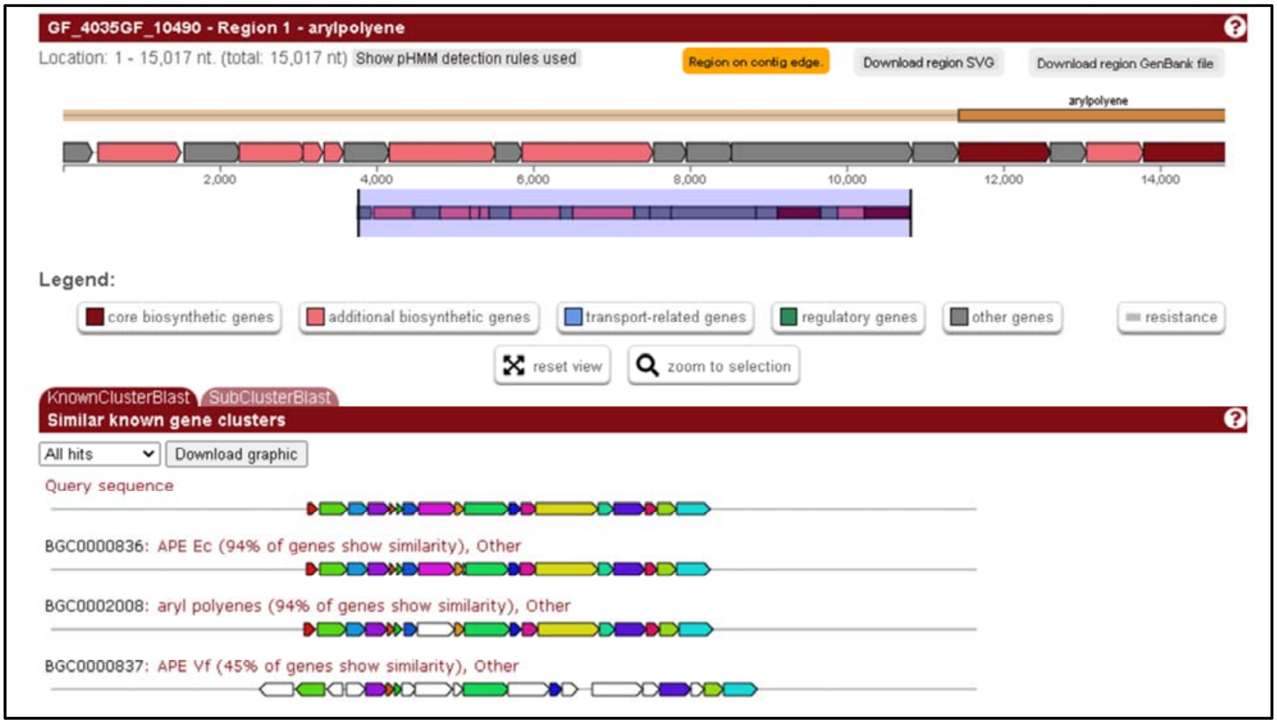

Supplementary Figure S15: Analyzing s2-ST11 with antiSMASH
antiSMASH result from analyzing s2-ST11. It is observed that the aryl polyene biosynthetic gene cluster is observed with 94% similarity. BGC0000836 is the biosynthetic cluster in the UPEC strain CFT073.

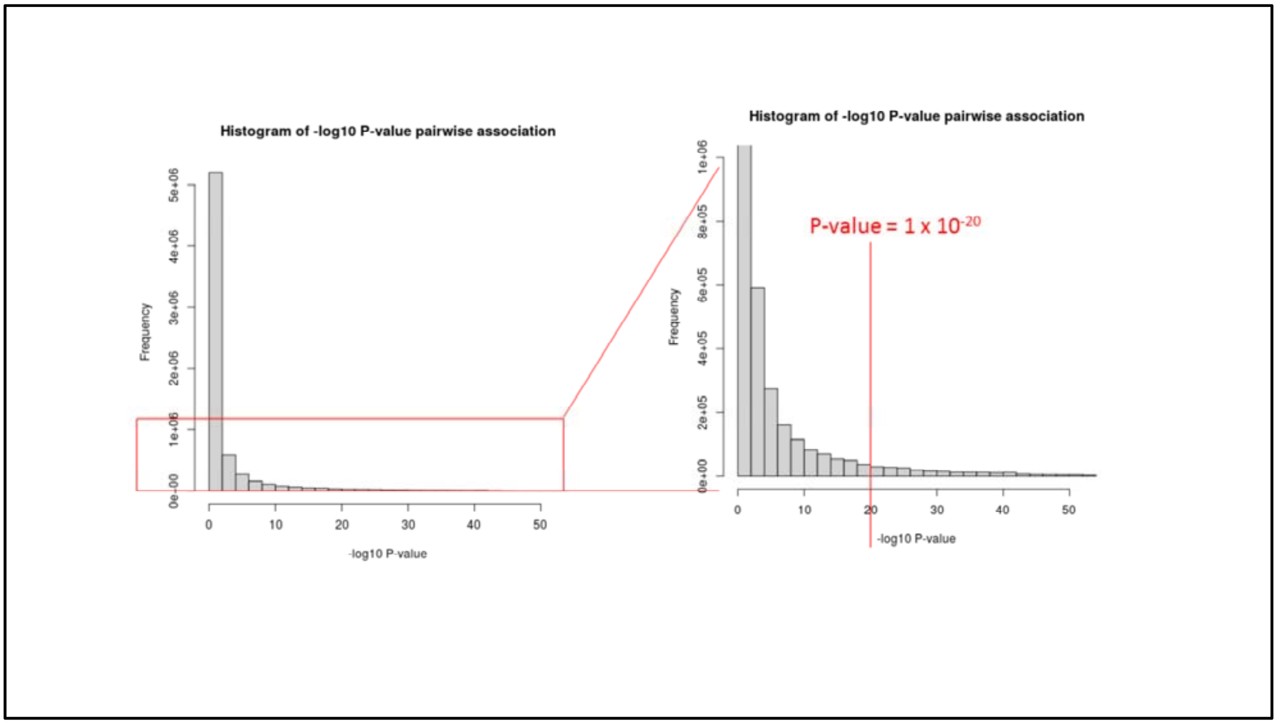

Supplementary Figure S16: Histogram of the -log10 P-value generated with CoinFinder
Histogram of the -log10 P-value of the pairwise GF association generated from the CoinFinder output for the all pairwise comparisons. The figure on the right is the enlarged section of the distribution with the y-axis truncated at 10^6. The P-value 1×10^{-20} is selected as the *ad hoc* cut-off criterion for significant pairwise comparisons in this study.

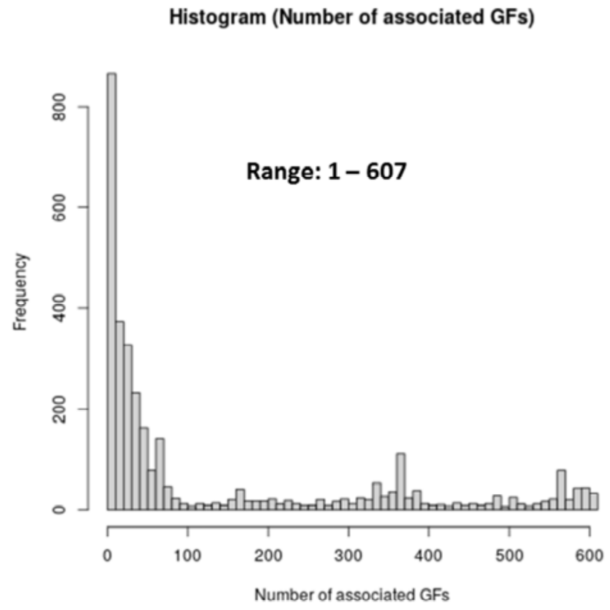

Supplementary Figure S17: Distribution of significantly associated GFs (associated GF cluster sizes)

The distribution of number of associated GFs for each significant GF (P-value <= 1×10^{-20}). The number of associated GFs for each significant GF ranges from 1 to 607. Though there are overwhelmingly high number of GFs with fewer than 50 associated GFs, there are quite a substantial number of GFs with many associated GFs as well, especially those with more than 300 associated GFs.
