# Supplementary material for: To kill or to be killed: pangenome analysis of Escherichia coli strains reveals a tailocin specific for pandemic ST131
Source: BMC Biol. 2022 Jun 16;20:146. doi: 10.1186/s12915-022-01347-7 (PMC9205054; doi:10.1186/s12915-022-01347-7)
Supplement: Supplementary file 2 — Additional file 2: Tables S1-S10. This Additional file 2 provides 10 supplementary tables supporting the conclusions in the main text. Tab. S1. Performance evaluation of the pangenome development. We list the numbers of clusters of homologous genes/proteins across different range of SeqID and SeqLC. Tab. S2. Effect of SeqID and SeqLC on the number of clusters. To evaluate the effect of SeqLC, we evaluate the number of clusters across different seqID at each SeqLC threshold using linear regression. The slope represents the amount of change with respect to every increase in SeqLC. Similarly, to evaluate the effect of SeqID, the number of clusters across different SeqLC threshold is evaluated and the slope is calculated. The evaluation is done on both methods, i.e., CD-HIT and ProteinOrtho. Tab. S3. Synteny clusters among the common genes specific to ST131 Escherichia coli. Tab. S4. Annotating the s1-ST131 cluster. Pseudomonas aeruginosa genes are represented with prefix PA. Tab. S5. Annotating the s2-ST131 cluster. Pseudomonas aeruginosa genes are represented with prefix PA. Tab. S6. Synteny clusters among the common genes specific to ST11 Escherichia coli. Tab. S7. The number of gene families (GFs) associated to s2-ST131 gene families. The identification of significantly associated gene families was carried out using CoinFinder based on a p-value <= 1-20 cutoff. Tab. S8. Supplementary Table S8: The number of gene families (GFs) associated to s1-ST11 gene families. The identification of significantly associated gene families was carried out using CoinFinder based on a p-value <= 1-20 cutoff. Tab. S9. Synteny cluster analysis of the GFs associated with s2-ST131. The inter-gene distance is kept at the maximum 1000 bp with at least 10 members per cluster. The s1-ST131 is excluded in this table. Tab. S10. Supplementary Table S10: Synteny cluster analysis of the GFs associated with s1-ST11. The inter-gene distance is kept at the maximum of 1000 bp with at least 10 members [file 12915_2022_1347_MOESM2_ESM.pdf]

Supplementary Table S1: Performance evaluation of the pangenome development

We list the numbers of clusters of homologous genes/proteins across different range of SeqID and SeqLC.

SeqID	SeqLC	CD-HIT	ProteinOrtho	Intersect	Jaccard Index
40	50	23852	22408	19199	70.95
40	60	24680	23608	20284	72.43
40	70	25671	25050	21353	72.71
40	80	27124	27239	22728	71.84
40	90	30034	30961	25160	70.21
50	50	27322	25644	23166	77.74
50	60	28083	26768	24053	78.10
50	70	29010	28097	24996	77.84
50	80	30344	30117	26207	76.51
50	90	33094	33515	28676	75.60
60	50	30749	28764	26471	80.11
**60**	**60**	**31465**	**29855**	**27370**	**80.62**
60	70	32365	31132	28327	80.54
60	80	33626	33039	29534	79.54
60	90	36220	36250	32030	79.20
70	50	34454	32069	29711	80.71
70	60	35140	33155	30712	81.72
70	70	35981	34484	31812	82.30
70	80	37215	36357	33131	81.92
70	90	39659	39400	35715	82.40
80	50	39190	36212	33599	80.37
80	60	39811	37382	34803	82.10
80	70	40612	38754	36128	83.56
80	80	41736	40619	37693	84.40
80	90	43973	43502	40496	86.20

Supplementary Table S2: Effect of SeqID and SeqLC on the number of clusters.

To evaluate the effect of SeqLC, we evaluate the number of clusters across different seqID at each SeqLC threshold using linear regression. The slope represents the amount of change with respect to every increase in SeqLC. Similarly, to evaluate the effect of SeqID, the number of clusters across different SeqLC threshold is evaluated and the slope is calculated. The evaluation is done on both methods, *i.e.*, CD-HIT and ProteinOrtho.

Evaluation	X-axis range	CD-HIT	ProteinOrtho
SeqID=40	SeqLC = 50, 60, 70, 80, 90	148.1	207.4
SeqID=50		138.1	190.9
SeqID=60		131.0	181.6
SeqID=70		124.9	178.6
SeqID=80		114.9	178.2
SeqLC=50	SeqID = 40, 50, 60, 70, 80	378.1	340.3
SeqLC=60		373.2	339.4
SeqLC=70		368.5	338.0
SeqLC=80		361.0	330.0
SeqLC=90		344.4	309.7

Supplementary Table S3: Synteny clusters among the common genes specific to ST131 *Escherichia coli*

**First Cluster: s1-ST131 (30 genes), DNA Length = 22,979 bp**		
**GF_8**	gene-SY51_RS04900	tail fiber protein
**GF_4394**	gene-SY51_RS04905	phage tail protein I
**GF_6067**	gene-SY51_RS04910	baseplate J/gp47 family protein
**GF_4923**	gene-SY51_RS04915	GPW/gp25 family protein
**GF_2624**	gene-SY51_RS04920	phage baseplate assembly protein V
**GF_3428**	gene-SY51_RS04925	phage late control D family protein
**GF_5265**	gene-SY51_RS04930	tail protein X
**GF_4614**	gene-SY51_RS04935	phage tail protein
**GF_515**	gene-SY51_RS04940	phage tail tape measure protein
**GF_3053**	gene-SY51_RS29995	GpE family phage tail protein
**GF_1673**	gene-SY51_RS04945	phage tail assembly protein
**GF_12285**	gene-SY51_RS04950	hypothetical protein
**GF_4965**	gene-SY51_RS04955	phage major tail tube protein
**GF_3382**	gene-SY51_RS04960	phage tail sheath subtilisin-like domain-containing protein
**GF_4777**	gene-SY51_RS04965	hypothetical protein
**GF_13685**	gene-SY51_RS04970	Gp37 family protein
**GF_2688**	gene-SY51_RS04975	DUF1320 domain-containing protein
**GF_1827**	gene-SY51_RS04980	DUF2190 family protein
**GF_3348**	gene-SY51_RS04985	hypothetical protein
**GF_5812**	gene-SY51_RS04990	hypothetical protein
**GF_13566**	gene-SY51_RS04995	phage virion morphogenesis protein
**GF_13563**	gene-SY51_RS05000	minor capsid protein
**GF_5811**	gene-SY51_RS05005	DUF935 domain-containing protein
**GF_4045**	gene-SY51_RS05010	hypothetical protein
**GF_2777**	gene-SY51_RS05015	DUF3486 family protein
**GF_13585**	gene-SY51_RS05020	hypothetical protein
**GF_19009**	gene-SY51_RS05025	hypothetical protein
**GF_1759**	gene-SY51_RS05030	hypothetical protein
**GF_1007**	gene-SY51_RS05035	transglycosylase SLT domain-containing protein
**GF_4864**	gene-SY51_RS05040	putative holin

**Second Cluster: s2-ST131 (33 genes), DNA Length = 23,818 bp**		
**GF_6212**	gene-SY51_RS10530	hypothetical protein
**DAS35886.1**	Corrected by homology	Late control protein (homology to AccID: BK034715.1)
**GF_13723**	gene-SY51_RS10540	tail protein X
**GF_12351**	gene-SY51_RS10545	phage tail protein
**GF_5820**	gene-SY51_RS10550	hypothetical protein
**GF_5060**	gene-SY51_RS10555	phage tail assembly protein

**GF_13643**	gene-SY51_RS10560	phage major tail tube protein
**GF_1688**	gene-SY51_RS10565	phage tail sheath subtilisin-like domain-containing protein
**GF_13623**	gene-SY51_RS10570	hypothetical protein
**GF_21864**	gene-SY51_RS10575	phage tail protein
**GF_13579**	gene-SY51_RS10580	phage tail protein I
**GF_13625**	gene-SY51_RS10585	baseplate J/gp47 family protein
**GF_13657**	gene-SY51_RS10590	GPW/gp25 family protein
**GF_22122**	gene-SY51_RS10595	phage baseplate assembly protein V
**GF_13550**	gene-SY51_RS10600	hypothetical protein
**GF_4850**	gene-SY51_RS10605	hypothetical protein
**GF_13725**	gene-SY51_RS10610	hypothetical protein
**GF_4345**	gene-SY51_RS10615	DNA-packaging protein
**GF_13706**	gene-SY51_RS10620	major capsid protein
**GF_13582**	gene-SY51_RS10625	hypothetical protein
**GF_21797**	gene-SY51_RS10630	S49 family peptidase
**GF_685**	gene-SY51_RS10635	phage portal protein
**GF_13580**	gene-SY51_RS10640	hypothetical protein
**GF_21794**	gene-SY51_RS10645	phage terminase large subunit family protein
**GF_13571**	gene-SY51_RS10650	hypothetical protein
**GF_13726**	gene-SY51_RS10655	hypothetical protein
**GF_4815**	gene-SY51_RS10660	hypothetical protein
**GF_2611**	gene-SY51_RS10665	TIGR02594 family protein
**GF_21804**	gene-SY51_RS10670	phage holin family protein
**GF_7242**	gene-SY51_RS10675	hypothetical protein
**GF_5648**	gene-SY51_RS10680	antitermination protein
**GF_800**	gene-SY51_RS10685	DUF1364 domain-containing protein
**GF_1507**	gene-SY51_RS10690	DUF1367 family protein
**GF_13672**	gene-SY51_RS10695	hypothetical protein

Supplementary Table S4: Annotating the s1-ST131 cluster.

Pseudomonas aeruginosa genes are represented with prefix PA

**GF_ID**	**Remarks**
**GF_8**	Short tail fiber protein gp12; T4, baseplate-tail tube complex (HHPRED)
**GF_4394**	PA0619
**GF_6067**	PA0618
**GF_4923**	PA0617
**GF_2624**	PA0616
**GF_3428**	PA0628
**GF_5265**	PA0627

**GF_4614**	Phage tail protein. PA0626?
**GF_515**	Phage tail tape measure protein. PA0625?
**GF_3053**	GpE family phage tail protein.
**GF_1673**	PA0624
**GF_12285**	hypothetical protein
**GF_4965**	PA0623
**GF_3382**	PA0622
**GF_4777**	hypothetical protein
**GF_13685**	PA0615 (HHPRED)
**GF_2688**	DUF1320, has coiled-coil segment, gp36 prophage protein
**GF_1827**	DUF2190, unknown function, has membrane embedded region, PTS. Capsid fiber protein (HHPRED)
**GF_3348**	Phage major capsid protein E; Major head protein (HHPRED)
**GF_5812**	Phage_GPO ; Phage capsid scaffolding protein (GPO) serine peptidase (HHPRED)
**GF_13566**	Phage_tail_S ; Phage virion morphogenesis family (HHPRED)
**GF_13563**	Phage_Mu_F ; Phage Mu protein F like protein (HHPRED)
**GF_5811**	Short tail fiber protein gp12; T4, baseplate-tail tube complex (HHPRED)
**GF_4045**	Terminase large subunit; genome packaging, bacteriophage, ATPase, nuclease (HHPRED)
**GF_2777**	DUF3486 family protein, unknown function
**GF_13585**	Ferric uptake regulation protein? (HHPRED)
**GF_19009**	Has TMM regions, PTS (ANNOTATOR). ATP synthase subunit b; Bacterial ATP synthase, HYDROLASE (HHPRED)
**GF_1759**	Has TMM regions, Coiled-coil, PTS (ANNOTATOR). Zinc resistance-associated protein, Salmonella typhimurium LT2 (HHPRED)
**GF_1007**	Has TMM regions, PTS, Lysozyme-like (ANNOTATOR). MEMBRANE-BOUND LYTIC MUREIN TRANSGLYCOSYLASE F (HHPRED)
**GF_4864**	Has TMM regions, PTS (ANNOTATOR). Holin_2-3 ; Putative 2/3 transmembrane domain holin (HHPRED)

Supplementary Table S5: Annotating the s2-ST131 cluster.

Pseudomonas aeruginosa genes are represented with prefix PA.

GF_ID	Remarks
**DAS35886.1**	PA0628
**GF_13723**	Pyocin Baseplate (glue) PA0627
**GF_12351**	Pyocin Baseplate (ripcord) PA0626
**GF_5820**	Not significant. Reannotate using BLASTP: DAS35881, tail tape measure: PA0625
**GF_5060**	Has TM segments (SAPS), chaperone protein, PA0624
**GF_13643**	Pyocin tube PA0623
**GF_1688**	Pyocin sheath PA0622

**GF_13623**	Not significant. Reannotate using BLASTP: Tail fiber assembly protein (WP_133521596.1). PA0621
**GF_21864**	Pyocin wedge, PA0620
**GF_13579**	Pyocin Tri2 PA0619
**GF_13625**	Pyocin Tri1a PA0618
**GF_13657**	Pyocin Sheath Initiator PA0617
**GF_22122**	Membrane piercing domain gp5, PA0616
**GF_13550**	Pyocin collar PA0615
**GF_4850**	Has TM segment, Prophage minor tail protein, PF06763.13
**GF_13725**	Has PTS signal, Phage Head-Tail Attachment, PF05354.13
**GF_4345**	Has coiled-coil segment, DNA Packaging protein, PF14000.8
**GF_13706**	Capsid protein of prophage, 3BQW_A
**GF_13582**	Has PTS signal, Head decoration protein, 1TD4_A
**GF_21797**	S49 family peptidase (Protease)
**GF_685**	Phage portal protein, GP20? PF05136.15, 6TE9_A, 3JA7_B
**GF_13580**	Has PTS signal, gpW, head-to-tail joining protein, PF02831, 2L6Q_A
**GF_21794**	Phage terminase large subunit, Gp17, PF05876.14, 2O0J_A
**GF_13571**	Phage DNA packaging protein Nu1, PF07471.14
**GF_13726**	Pyocin-S2 immunity protein; HNH Nuclease Domain (HHPRED)
**GF_4815**	Outer membrane lipoprotein (HHPRED); lysis protein (BLASTP)
**GF_2611**	Lysin; CHAP-domain (may involve in peptidoglycan hydrolysis)
**GF_21804**	Putative 3TM Holin (PF05449)
**GF_7242**	Putative Phage Holin (PF16931)
**GF_5648**	Coiled-coil segment; bacteriophage antitermination protein Q
**GF_800**	PF07102 (unknown function), zinc-binding, His-Me superfamily which is important in defense and stress response. DUF1364
**GF_1507**	PF07105 (unknown function), several proteins in this family are annotated as IrsA (intracellular response to stress). DUF1367
**GF_13672**	Nif11-domain (BLASTP), PF07862 (N11P sequences have a classic leader peptide cleavage motif, usually Gly-Gly)

Supplementary Table S6: Synteny clusters among the common genes specific to ST11 *Escherichia coli*

**First Cluster: s1-ST11 (16 genes), DNA Length = 19,347 bp**		
**GF_10888**	ECs_1282	hemagglutinin/hemolysin-related protein
**GF_10889**	ECs_1283	hemolysin activator-related protein
**GF_10890**	ECs_1284	holo-[acyl-carrier protein] synthase
**GF_10891**	ECs_1285	3-oxoacyl-[acyl-carrier protein] reductase
**GF_10892**	ECs_1286	3-hydroxyacyl-[acyl-carrier-protein] dehydratase
**GF_10893**	ECs_1287	acyl carrier protein
**GF_10894**	ECs_1288	aminomethyltransferase
**GF_10895**	ECs_1289	3-oxoacyl-[acyl-carrier-protein] synthase synthase

**GF_10896**	ECs_1290	beta-ketoacyl synthase
**GF_10897**	ECs_1291	hypothetical protein
**GF_10898**	ECs_1292	ABC transporter ATP-binding protein
**GF_10899**	ECs_1293	hypothetical protein
**GF_10900**	ECs_1294	ABC transport system permease protein
**GF_10901**	ECs_1295	hypothetical protein
**GF_10902**	ECs_1296	hypothetical protein
**GF_10903**	ECs_1297	hypothetical protein

**Second Cluster: s2-ST11 (18 genes), DNA Length = 15,017 bp**		
**GF_4035**	ECs_4324	putative lipoprotein
**GF_10509**	ECs_4325	O-methyltransferase
**GF_10722**	ECs_4326	hypothetical protein
**GF_10765**	ECs_4327	acyltransferase
**GF_10665**	ECs_4328	acyl carrier protein
**GF_10474**	ECs_4329	acyl carrier protein
**GF_2429**	ECs_4330	DNA gyrase subunit B
**GF_8078**	ECs_4331	surfactin synthetase
**GF_5318**	ECs_4332	(3R)-hydroxymyristoyl-[acyl carrier protein] dehydratase
**GF_10469**	ECs_4333	acyltransferase
**GF_10617**	ECs_4334	acyl-CoA thioester hydrolase
**GF_10571**	ECs_4335	outer membrane lipoprotein carrier protein LolA
**GF_23585**	ECs_4336	hypothetical protein
**GF_10560**	ECs_4337	lipoprotein
**GF_10723**	ECs_4338	3-oxoacyl-(acyl-carrier-protein) synthase II
**GF_10443**	ECs_4339	beta-hydroxydecanoyl-ACP dehydrase
**GF_10462**	ECs_4340	3-oxoacyl-[acyl-carrier-protein] reductase
**GF_10490**	ECs_4341	3-oxoacyl-(acyl-carrier-protein) synthase II

Supplementary Table S7: The number of gene families (GFs) associated to s2-ST131 gene families

The identification of significantly associated gene families was carried out using CoinFinder based on a p-value <= 1^{-20} cutoff.

**GF_ID**	**#Associated GFs**
GF_6212	365
GF_13723	365
GF_12351	364
GF_5820	366
GF_5060	367
GF_13643	367
GF_1688	364
GF_13623	369

GF_21864	367
GF_13579	367
GF_13625	364
GF_13657	364
GF_22122	362
GF_13550	367
GF_4850	367
GF_13725	367
GF_4345	367
GF_13706	364
GF_13582	213
GF_21797	367
GF_685	0 (p-value > 1e-20)
GF_13580	367
GF_21794	323
GF_13571	364
GF_13726	367
GF_4815	367
GF_2611	328
GF_21804	362
GF_7242	362
GF_5648	366
GF_800	0 (p-value > 1e-20)
GF_1507	0 (p-value > 1e-20)
GF_13672	367

Supplementary Table S8: The number of gene families (GFs) associated to s1-ST11 gene families

The identification of significantly associated gene families was carried out using CoinFinder based on a p-value <= 1^{-20} cutoff.

GF_ID	#Associated GFs
GF_10888	583
GF_10889	590
GF_10890	594
GF_10891	594
GF_10892	594
GF_10893	590
GF_10894	594
GF_10895	591
GF_10896	591
GF_10897	589
GF_10898	589
GF_10899	591

GF_10900	591
GF_10901	588
GF_10902	591
GF_10903	588

Supplementary Table S9: Synteny cluster analysis of the GFs associated with s2-ST131

The inter-gene distance is kept at the maximum 1000 bp with at least 10 members per cluster. The s1-ST131 is excluded in this table.

**First Cluster: 10 gene families**		
**GF_11236**	flhB	flagellar type III secretion system protein FlhB
**GF_11329**	fliR	flagellar biosynthetic protein FliR
**GF_11268**	fliQ	flagellar type III secretion system protein FliQ
**GF_11108**	SY51_RS01275	FliM/FliN family flagellar motor switch protein
**GF_4979**	SY51_RS01280	FliM/FliN family flagellar motor switch protein
**GF_11333**	SY51_RS01285	sigma-54-dependent Fis family transcriptional regulator
**GF_8162**	SY51_RS01290	flagellar hook-basal body complex protein FliE
**GF_27076**	fliF	flagellar M-ring protein FliF
**GF_3121**	SY51_RS01300	flagellar motor switch protein FliG
**GF_6904**	SY51_RS01305	flagellar assembly protein H

**Second Cluster: 13 gene families**		
**GF_11253**	SY51_RS01400	rod-binding protein
**GF_28272**	flgK	flagellar hook-associated protein FlgK
**GF_11331**	flgL	flagellar hook-associated protein FlgL
**GF_11314**	SY51_RS01415	hypothetical protein
**GF_7185**	SY51_RS01420	winged helix-turn-helix domain-containing protein
**GF_11266**	lafA	lateral flagellin LafA
**GF_4725**	fliD	flagellar filament capping protein FliD
**GF_11155**	fliS	flagellar export chaperone FliS
**GF_2723**	SY51_RS01440	hypothetical protein
**GF_16855**	SY51_RS01445	flagellar hook-length control protein FliK
**GF_11225**	SY51_RS01450	flagellar basal body-associated FliL family protein
**GF_11265**	SY51_RS01455	FliA/WhiG family RNA polymerase sigma factor
**GF_13775**	motA	flagellar motor stator protein MotA

**Third Cluster: 14 gene families**		
**GF_14930**	tssK	type VI secretion system baseplate subunit TssK
**GF_14941**	tssL	type VI secretion system protein TssL%2C short form
**GF_3089**	SY51_RS15765	OmpA family protein
**GF_2964**	SY51_RS15770	Hcp family type VI secretion system effector
**GF_2622**	tssH	type VI secretion system ATPase TssH
**GF_3853**	vgrG	type VI secretion system tip protein VgrG
**GF_13620**	SY51_RS15785	hypothetical protein
**GF_13727**	SY51_RS15790	hypothetical protein
**GF_20629**	SY51_RS15800	DUF4123 domain-containing protein
**GF_1820**	SY51_RS15805	DUF3304 domain-containing protein

**GF_3230**	SY51_RS15810	DUF2235 domain-containing protein
**GF_4836**	SY51_RS15820	transglycosylase SLT domain-containing protein
**GF_15350**	SY51_RS15825	hypothetical protein
**GF_5169**	SY51_RS15830	hypothetical protein
**Fourth Cluster: 27 gene families**		
**GF_2777**	SY51_RS05015	DUF3486 family protein
**GF_13585**	SY51_RS05020	hypothetical protein
**GF_19009**	SY51_RS05025	hypothetical protein
**GF_1759**	SY51_RS05030	hypothetical protein
**GF_1007**	SY51_RS05035	transglycosylase SLT domain-containing protein
**GF_4864**	SY51_RS05040	putative holin
**GF_2626**	SY51_RS05045	hypothetical protein
**GF_13711**	SY51_RS05050	hypothetical protein
**GF_5115**	SY51_RS05055	hypothetical protein
**GF_2829**	SY51_RS05060	DNA adenine methylase
**GF_13552**	SY51_RS05065	helix-turn-helix transcriptional regulator
**GF_1700**	SY51_RS05070	DNA-binding protein
**GF_4288**	SY51_RS05075	helix-turn-helix domain-containing protein
**GF_428**	SY51_RS05080	DUF3102 domain-containing protein
**GF_13682**	SY51_RS05085	hypothetical protein
**GF_6948**	SY51_RS05090	DDE-type integrase/transposase/recombinase
**GF_13666**	SY51_RS05095	AAA family ATPase
**GF_21098**	SY51_RS05100	hypothetical protein
**GF_11056**	SY51_RS05105	host-nuclease inhibitor Gam family protein
**GF_1849**	SY51_RS05115	hypothetical protein
**GF_4447**	SY51_RS05120	hypothetical protein
**GF_13638**	SY51_RS05125	hypothetical protein
**GF_4382**	SY51_RS05130	DUF2786 domain-containing protein
**GF_4745**	SY51_RS05135	hypothetical protein
**GF_13581**	SY51_RS05140	hypothetical protein
**GF_13692**	SY51_RS05145	regulatory protein GemA
**GF_3113**	SY51_RS05150	DNA-binding protein
**Fifth Cluster: 11 gene families**		
**GF_13698**	SY51_RS10735	DUF1311 domain-containing protein
**GF_3555**	SY51_RS10740	ORF6N domain-containing protein
**GF_4772**	SY51_RS10750	hypothetical protein
**GF_13659**	SY51_RS10760	hypothetical protein
**GF_13584**	SY51_RS10765	hypothetical protein
**GF_4724**	SY51_RS10770	AAA family ATPase

**GF_13670**	SY51_RS10775	hypothetical protein
**GF_4997**	SY51_RS10780	hypothetical protein
**GF_13599**	SY51_RS10785	hypothetical protein
**GF_3458**	SY51_RS10790	helix-turn-helix domain-containing protein
**GF_13713**	SY51_RS10795	helix-turn-helix domain-containing protein

Supplementary Table S10: Synteny cluster analysis of the GFs associated with s1-ST11

The inter-gene distance is kept at the maximum of 1000 bp with at least 10 members per cluster. The s2-ST11 cluster is excluded in this table.

**First Cluster: 12 gene families**		
**GF_20171**	ECs_0296	transcription activator
**GF_25895**	ECs_0297	phage polarity suppression protein
**GF_1561**	ECs_0298	phage capsid size determining protein
**GF_397**	ECs_0299	transcriptional regulator
**GF_388**	ECs_0300	phage repressor protein
**GF_1455**	ECs_0301	hypothetical protein
**GF_398**	ECs_0302	hypothetical protein
**GF_486**	ECs_0303	phage DNA primase
**GF_11144**	ECs_0304	hypothetical protein
**GF_10826**	yagP	LysR family transcriptional regulator
**GF_10827**	ECs_0306	oxidoreductase
**GF_1562**	ECs_0307	putative membrane protein

**Second Cluster: 19 gene families**		
**GF_10875**	ECs_1223	phage major capsid protein
**GF_3047**	ECs_1224	hypothetical protein
**GF_3989**	ECs_1225	hypothetical protein
**GF_3990**	ECs_1226	hypothetical protein
**GF_3991**	ECs_1227	hypothetical protein
**GF_98**	ECs_1228	phage tail fiber protein
**GF_47**	ECs_3489	hypothetical protein
**GF_10876**	ECs_1230	hypothetical protein
**GF_5170**	ECs_1232	hypothetical protein
**GF_1718**	ECs_1233	phage tail fiber protein
**GF_2209**	ECs_1234	outer membrane protein
**GF_1114**	ECs_1235	hypothetical protein
**GF_833**	ECs_1236	outer membrane precursor protein Lom
**GF_3992**	ECs_1237	hypothetical protein
**GF_10877**	ECs_1238	hypothetical protein
**GF_10878**	ECs_1239	hypothetical protein

**GF_1572**	ECs_1240	hypothetical protein
**GF_643**	ECs_1241	hypothetical protein
**GF_3492**	ECs_1242	hypothetical protein

**Third Cluster: 10 gene families**		
**GF_10882**	ECs_1272	diguanylate phosphodiesterase
**GF_19414**	ECs_1273	FidL-like protein
**GF_27132**	grvA	transcriptional regulator
**GF_10883**	ECs_1275	oxidoreductase
**GF_10884**	ECs_1276	chaperone protein
**GF_10885**	ECs_1277	outer membrane protein
**GF_26644**	ECs_1278	outer membrane usher protein
**GF_26645**	ECs_1279	chaperone protein
**GF_10887**	ECs_1280	major pilin protein
**GF_4753**	ECs_1281	hypothetical protein

**Fourth Cluster: 14 gene families**		
**GF_401**	ECs_1341	hypothetical protein
**GF_1178**	ECs_1342	hypothetical protein
**GF_3997**	terW	tellurium resistance protein TerW
**GF_3998**	ECs_1344	hypothetical protein
**GF_3999**	ECs_1345	hypothetical protein
**GF_4000**	ECs_1346	hypothetical protein
**GF_5720**	ECs_1348	hypothetical protein
**GF_6517**	ECs_1349	hypothetical protein
**GF_4001**	ECs_1350	hypothetical protein
**GF_4002**	terE	tellurium resistance protein TerE
**GF_6516**	terA	tellurium resistance protein TerA
**GF_10912**	terB	tellurium resistance protein TerB
**GF_10913**	terC	tellurium resistance protein TerC
**GF_4003**	terF	tellurium resistance protein TerF

**Fifth Cluster: 13 gene families**		
**GF_929**	ECs_1503	exodeoxyribonuclease VIII
**GF_644**	ECs_1504	hypothetical protein
**GF_24415**	ECs_1506	phage repressor
**GF_2464**	ydaS	antirepressor
**GF_1399**	ECs_2763	replication protein
**GF_838**	ECs_2762	phage replication protein
**GF_147**	ECs_2203	hypothetical protein
**GF_329**	ECs_1514	hypothetical protein

**GF_2396**	ECs_1515	hypothetical protein
**GF_11584**	ECs_1516	hypothetical protein
**GF_14204**	ECs_1517	hypothetical protein
**GF_3845**	ECs_1518	putative phage protein
**GF_10952**	ECs_2755	hypothetical protein

**Sixth Cluster: 13 gene families**		
**GF_588**	ECs_2967	phage antirepressor
**GF_18807**	ECs_5741	hypothetical protein
**GF_331**	ECs_1536	hypothetical protein
**GF_13471**	ECs_1538	hypothetical protein
**GF_547**	ECs_2254	DNase
**GF_645**	ECs_2252	phage terminase small subunit
**GF_805**	ECs_2251	phage terminase large subunit
**GF_6468**	ECs_2250	phage prohead protease
**GF_484**	ECs_5451	hypothetical protein
**GF_791**	ECs_2248	phage portal protein
**GF_17360**	ECs_2247	phage DNA packaging protein
**GF_3770**	ECs_2246	phage head-tail adaptor
**GF_1078**	ECs_2244	phage minor tail protein

**Seventh Cluster: 10 gene families**		
**GF_10952**	ECs_2755	hypothetical protein
**GF_16174**	ECs_2756	hypothetical protein
**GF_404**	ECs_2757	hypothetical protein
**GF_1597**	ECs_4958	hypothetical protein
**GF_480**	ECs_2759	hypothetical protein
**GF_8111**	ECs_2760	hypothetical protein
**GF_838**	ECs_2762	phage replication protein
**GF_1399**	ECs_2763	replication protein
**GF_1213**	ECs_2765	phage antirepressor protein Cro
**GF_10954**	ECs_2766	phage repressor protein CI

**Eight Cluster: 17 gene families**		
**GF_165**	espF	T3SS secreted effector EspF
**GF_2434**	escG	T3SS component EscG
**GF_11019**	escF	T3SS structure protein EscF
**GF_11020**	cesD	T3SS chaperone CesD2
**GF_1132**	espB	T3SS translocator EspB
**GF_848**	espD	T3SS translocator EspD
**GF_849**	espA	T3SS translocator EspA

**GF_11021**	sepL	T3SS secretion switching protein SepL
**GF_11022**	escD	T3SS structure protein EscD
**GF_337**	eae	T3SS intimin
**GF_11023**	cesT	T3SS chaperone CesT
**GF_759**	tir	T3SS translocated intimin receptor Tir
**GF_717**	map	T3SS secreted effector Map
**GF_1592**	cesF	T3SS chaperone CesF
**GF_1133**	espH	T3SS secreted effector EspH
**GF_2996**	sepQ	T3SS structure protein SepQ
**GF_11024**	ECs_4567	T3SS component

**Ninth Cluster: 19 gene families**		
**GF_6406**	escV	T3SS structure protein EscV
**GF_11027**	mpc	regulator Mpc
**GF_496**	espZ	T3SS secreted effector EspZ
**GF_11028**	rorf8	T3SS component
**GF_11029**	escJ	T3SS structure protein EscJ
**GF_11030**	sepD	T3SS secretion switching protein SepD
**GF_11031**	escC	T3SS structure protein EscC
**GF_11032**	cesD	T3SS chaperone CesD
**GF_11033**	grlA	positive regulator GrlA
**GF_2435**	grlR	negative regulator GrlR
**GF_4805**	ipgF	hypothetical protein
**GF_11034**	escU	T3SS structure protein EscU
**GF_11035**	escT	T3SS structure protein EscT
**GF_11036**	escS	T3SS structure protein EscS
**GF_1593**	ECs_4584	T3SS component
**GF_11038**	ECs_4585	T3SS component
**GF_4325**	ECs_4586	T3SS component
**GF_11039**	ECs_4587	T3SS component
**GF_11040**	ler	transcription regulator Ler
