# Supplementary material for: To kill or to be killed: pangenome analysis of Escherichia coli strains reveals a tailocin specific for pandemic ST131
Source: BMC Biol. 2022 Jun 16;20:146. doi: 10.1186/s12915-022-01347-7 (PMC9205054; doi:10.1186/s12915-022-01347-7)
Supplement: Supplementary file 3 — Additional file 3. The Additional file 3 is a compressed file library (zip package) containing 11 files. File 1 genome list with serotypes, sequence types, phylogroups, etc. File 2A pangenome matrix determined with CD-HIT. File 2B softcore genome GF list determined with CD-HIT. File 3A pangenome matrix determined with ProteinOrtho. File 3B softcore genome GF list determined with ProteinOrtho. File 4 the GFs of the six distinctive clusters. File 5 summary of the strains’ virulence and antibiotics resistance. File 6 antibiogram data. File 7 virulence factor PAM. File 8 coincident pairwise association results from CoinFinder. README. [file 12915_2022_1347_MOESM3_ESM.zip › Additional File 3/Readme - Supplementary Files.docx]

**List of supplementary files in Additional File 3:**

Supplementary File 1.txt

Dataset characteristics of the 1,624 *E. coli* complete genomes. This includes NumChromosomal (number of chromosomal sequences), NumPlasmid (number of plasmid sequences), NumExtraChromosomal (number of extra chromosomal sequences), Length_DNA (length of DNA sequences), NumProteins (number of protein sequences), GC (GC content), SequenceType, Phylogroup, Serotype and Remarks.

Supplementary File 2A.txt

Pangenome matrix of *E. coli* based on CD-HIT method. The gene families (GFs) are represented on rows and the genomes are represented on columns. There are 25,420 GFs with 1,324 genomes. The entry 1 represents presence and 0 represents absence.

Supplementary File 2B.txt

The list of gene families (GFs) which belong to the softcore genome of *E. coli* identified through CD-HIT method.

Supplementary File 3A.txt

Pangenome matrix of *E. coli* based on ProteinOrtho method. The gene families (GFs) are represented on rows and the genomes are represented on columns. There are 24,889 GFs with 1,324 genomes. The entry 1 represents presence and 0 represents absence.

Supplementary File 3B.txt

The list of gene families (GFs) which belong to the softcore genome of *E. coli* identified through ProteinOrtho method.

Supplementary File 4.xlsx

The list of gene families (GFs) selected which show distinctive profile in different sequence types and phylotypes of *E. coli*. There are six worksheets in the file which can be categorized into (1) Rare in ST11; (2) Common in B2; (3) Common in ST131; (4) Rare in ST131; (5) B1-Shiga; and (6) Common in ST11. The list of GFs are given together with the gene name as well as description accordingly.

Supplementary File 5.txt

The number of virulence factors (Count_Virulence) and antibiotic resistant genes (Count_Antibiotic) present in the 1,324 *E. coli* genomes. The sequence type and phylogroup of each genome is given to facilitate analysis. The likelihood of pathogenicity is given by the column VirulenceCategory, with the value: 1 – likely nonpathogenic, 2 – likely virulence, 3 – high virulence and 4 – very high virulence.

Supplementary File 6.txt

The antibiogram matrix (24 antibiotics) with 1 represent resistant and 0 represent sensitive as predicted by ResFinder v4.1 for the 1,324 *E. coli* genomes.

Supplementary File 7.txt

The virulence factor matrix (177 virulence factors) with 1 represents presence and 0 represents absence as predicted by virulencefinder v2.0.3 for the 1,324 *E. coli* genomes.

Supplementary File 8.txt

The CoinFinder pairwise association for 674 genomes where the pairwise association p-value is smaller than 1 x 10^-20^
