# Supplementary material for: To kill or to be killed: pangenome analysis of Escherichia coli strains reveals a tailocin specific for pandemic ST131
Source: BMC Biol. 2022 Jun 16;20:146. doi: 10.1186/s12915-022-01347-7 (PMC9205054; doi:10.1186/s12915-022-01347-7)
Supplement: Supplementary file 4 — Additional file 4: Supplementary Methods. The Supplementary Methods file is available both at GitHub https://github.com/biierwint/ecoli_pangenome [180] as well as Additional file 4 with this article. [file 12915_2022_1347_MOESM4_ESM.pdf]

Additional File 4 - Supplementary Methods
for the article:

To kill or to be killed - pangenome analysis of *Escherichia coli* strains reveals a tailocin specific for pandemic ST131

Erwin Tantoso (1,2), Birgit Eisenhaber (1,2), Miles Kirsch (2,&), Vladimir Shitov (2), Zhiya Zhao (2,#) and Frank Eisenhaber (1,2,3,$)

1 Genome Institute of Singapore (GIS), Agency for Science, Technology and Research (A*STAR), 60 Biopolis Street, Singapore 138672, Republic of Singapore

2 Bioinformatics Institute (BII), Agency for Science, Technology and Research (A*STAR), 30 Biopolis Street #07-01, Matrix Building, Singapore 138671, Republic of Singapore

3 Department of Biological Sciences, National University of Singapore, 16 Science Drive 4, 117558 Singapore, Republic of Singapore

$ corresponding author
& present address: Northeastern University, United States of America
# present address: The University of Cambridge, Cambridge, United Kingdom of Great Britain and Northern Ireland

Emails: erwint@bii.a-star.edu.sg, birgite@bii.a-star.edu.sg, mileskirsch@gmail.com, vladimirshitov98@gmail.com, zz421@cam.ac.uk, franke@bii.a-star.edu.sg

In summary, we have provided:
a) The detailed steps on preparing E. coli genomes for analysis; as well as all the E. coli genomes available in NCBI Refseq as at 10th June 2021
b) All the necessary scripts and programs’ reference as per needed to do the analysis
c) The detailed steps for performing ANI pairwise calculation and selecting genomes for analysis
d) The detail steps on finding homologous clusters (Gene Family clusters)
e) The detail information on how to obtain the softcore genome
f) The command lines on how to execute MLST, SerotypeFinder, VirulenceFinder and ResFinder to obtain the MLST, serotype, virulence factor and antibiotic resistance typing
g) The command lines on the coincidence analysis

Preparing Escherichia coli genomes for analysis

(1) Selecting Escherichia coli species.
From the NCBI assembly_summary_refseq.txt downloaded on 10th June 2021, we extracted the assembly accession IDs if the column "organism_name" contained "Escherichia coli". This resulted in 23547 unique assembly accession IDs (misc-data/Ecoli-assembly_summary_refseq_10June2021.txt).

(2) Selecting assembly_accessions with complete genomes.
For our study, we concentrate on complete E.coli genomes. Therefore, in addition to the "organism_name" containing Escherichia coli, we need the

criterion "assembly_level" = Complete Genome to be fulfilled. This reduced the number of unique assembly accession IDs to 1626.

(3) Excluding phage genomes and assemblies with different sequences in RefSeq and GenBank.
From the column "organism_name", we noticed that two genome assemblies belong to E. coli O157 typing phages 3 and 6, respectively (GCF_002604825.1 and GCF_002604865.1).
From the column "paired_asm_comp", we further noticed that two genome assemblies (GCF_000184185.1 and GCF_002925525.1) have "different" sequences in RefSeq and GenBank.
The four assembly accession IDs were excluded from our analysis. This resulted in the final set of 1622 complete E.coli genomes (with 1622 unique assembly accession IDs: SupplementaryFile1.txt).

Setting up scripts and programs

We provide the codes used in the "scripts" folder.
Please note that the codes are tested on Ubuntu 18.04.3 LTS.

Prerequisite:
- Perl 5, version 26, subversion 1 (v5.26.1)

The "scripts/perl-lib" folder contains the PERL libraries necessary to run the scripts. Please add the PERL5LIB to your $HOME/.profile.

```
export PERL5LIB=$PERL5LIB:/path/to/scripts/perl-lib/
```

The "scripts/perl-scripts" folder contains the PERL scripts to carry out our analysis. The folder can be added to your $HOME/.profile (if necessary).

```
export PATH=$PATH:/path/to/scripts/perl-scripts:.
```

Further external programs required (please install the programs following the instructions from the respective webpage):
- fastANI: https://github.com/ParBLiSS/FastANI (version 1.32)
- CD-HIT: https://github.com/weizhongli/cdhit (version 4.7 built on Jul 1 2017)
- ProteinOrtho: https://gitlab.com/paulklemm_PHD/proteinortho (version 6.0.27)
- roary: https://sanger-pathogens.github.io/Roary/ (version 3.11.2)
- MLST: https://cge.cbs.dtu.dk/services/MLST/ (software version 2.0.4; database version 2.0.0)
- SerotypeFinder: https://cge.cbs.dtu.dk/services/SerotypeFinder/ (software version 2.0.1; database version 1.0.0)
- VirulenceFinder: https://cge.cbs.dtu.dk/services/VirulenceFinder/ (software version 2.0.3; database 2020-05-29)
- ResFinder: https://cge.cbs.dtu.dk/services/ResFinder/ (software version 4.1; database 2021-08-16)
- MUSCLE v3.8.31: https://drive5.com/muscle/downloads_v3.htm
- snp-sites: https://github.com/sanger-pathogens/snp-sites
- raxml v8.2.11 (with SSE3 support): https://cme.h-its.org/exelixis/web/software/raxml/
- CoinFinder: https://github.com/fwhelan/coinfinder

To remove almost identical genomic sequences from the analysis, which in turn reduces redundancy in our dataset, we used fastANI (version 1.32 with default parameters) to calculate the pairwise average nucleotide

identity (ANI) of all the 1,622 genomes. If the pairwise ANI is greater than 99.99%, then the genome with larger size was kept and the smaller genome was excluded from the analysis. This step reduced our dataset from 1622 to 1324 complete genomes.
We have prepared a batch script to process this step:
(1) run-fastANI-1622genomes.sh (To run in parallel: scripts/perl-scripts/distribute-jobs -i run-fastANI-1622genomes.sh)
This will create a folder called "output/".
(2) Run the script "scripts/perl-scripts/samples-selection-fastANI"
perl scripts/perl-scripts/samples-selection-fastANI -i misc-data/1622genomes-ordered.txt -f output -t 99.99
"-i misc-data/1622genomes-ordered.txt" should be ordered according to genome size, except for the first one which is our reference genome, i.e. K-12 MG1655.
"-f output" is the output folder that contains the fastANI output
"-t 99.99" is the threshold (99.99%) to consider that two genomes are very similar to each other.
As a result, the selected genomes will be printed to the screen and a separate file named "dropsamples.txt" is created which contains the details of which genomes are excluded.

Finding clusters of homologous sequences (Gene Families):
1. For each genome: finding almost identical protein sequences within the genome at SeqID=98% and SeqLC=100% and then select the longest protein sequence as representative sequence.

- ⇨ perl scripts/perl-scripts/preprocess-fasta -i list-of-proteome-files.txt -x -m cdhit -s 98 -l 100

[Note: This step will produce the file: "merged.faa" and the folder: "AIS". The file "merged.faa" contains all unique protein sequences from all genomes (almost identical sequences found in each of the genomes have been excluded). Plese make a copy of this file because it will be used/changed later. The "AIS" folder contains the almost identical sequence IDs from each genome with the first column as the ID of the representative sequence. Concatenate all files inside "AIS" folder to get the file "all-within-genome.AIS"]

2. Finding the core genome with at least 98% SeqID across all the genomes using the program iterative_cdhit (from Roary package)

- ⇨ iterative_cdhit -m merged.faa -n 1324 -l 98.0 -s 0.5

[Note: This will produce two files: merged.faa and "merged.faa.groups". The file "merged.faa.groups" contains the protein sequence IDs of the core genome. The file "merged.faa" contains all protein sequences which are not part of the core genome. Note that the file "merged.faa.groups" will be required later for the reinflating procedure to get the final clusters.

3. Finding homologous protein sequences at SeqID=90% and SeqLC=90% from merged.faa and then select the longest protein sequence as representative sequence.

- ⇨ cd-hit -i merged.faa -c 0.90 -s 0.90 -n 5 -o cdhit-c0.90-s0.90.out -d 50

[Note: The output cdhit-c0.90-s0.90.out will be used to iteratively find the best parameters for detecting clusters of homologous genes (gene families). Renamed this file to 52798sequences.faa. Note that these 52798 selected representative sequences belong to only 1272 genomes. The protein sequences from each of the remaining 52 genomes can be represented by the selected 52798 sequences. As a result, we will create

two files: 1272genomes.txt and 52additionalGenomes.txt. Both files contain the respective genome IDs.
- Reformating the CD-HIT cdhit-c0.90-s0.90.out.clst output (cluster file) The IDs of the 52798 representative sequences are in the first column.
[Note: use scripts/misc-scripts/get-id90-cov90-list.sh]

4. Running CD-HIT and ProteinOrtho for each combination of SeqID (40-80%) and SeqLC (50-90%)
For example, for CD-HIT: SeqID=60, SeqLC=60, word_length=4
- ⇨ cd-hit -i 52798sequences.faa -c 0.60 -s 0.60 -n 3 -d 50 -M 16000 -o cdhit-c60-s60.out
- ⇨ scripts/perl-scripts/cdhit2proteinortho -i cdhit-c60-s60.out -l misc-data/1272genomes.txt > output-cdhit-id60-cov60.txt

reinflate clusters
- ⇨ scripts/perl-scripts/merge-clusters -i output-cdhit-id60-cov60.txt -l mylist-id90-cov90.txt -g misc-data/52additionalGenomes.txt
- ⇨ scripts/perl-scripts/reinflate-clusters -i output-cdhit-id60-cov60.txt-remerged -l merged.faa.groups
- ⇨ scripts/perl-scripts/merge-clusters -i output-cdhit-id60-cov60.txt-remerged-reinflate -l all-within-genome.AIS
- ⇨ mv output-cdhit-id60-cov60.txt-remerged-reinflate-remerged final-output-cdhit-id60-cov60.txt

Example for ProteinOrtho: SeqID=60, SeqLC=60
- ⇨ proteinortho_nobest -p=blastp+ -identity=60 -cov=60 -selfblast -mcl -project=myproject-id60-cov60 52798sequences.faa
- ⇨ scripts/perl-scripts/selfblast2proteinortho -i myproject-id60-cov60.proteinortho.tsv -l misc-data/1272genomes.txt > output-proteinortho-id60-cov60.txt
- ⇨ scripts/perl-scripts/add-singletons -i output-proteinortho-id60-cov60.txt -l allproteins.txt [Note: allproteins.txt is the protein ID from the 52798sequences.faa]

reinflate clusters
- ⇨ scripts/perl-scripts/merge-clusters -i output-proteinortho-id60-cov60.txt-all -l mylist-id90-cov90.txt -g misc-data/52additionalGenomes.txt
- ⇨ scripts/perl-scripts/reinflate-clusters -i output-proteinortho-id60-cov60.txt-all-remerged -l merged.faa.groups
- ⇨ scripts/perl-scripts/merge-clusters -i output-proteinortho-id60-cov60.txt-all-remerged-reinflate -l all-within-genome.AIS
- ⇨ mv output-proteinortho-id60-cov60.txt-all-remerged-reinflate-remerged final-output-proteinortho-id60-cov60.txt

5. Run reclustering
- ⇨ scripts/misc-scripts/run-recluster-CD-HIT.sh [This will produce: recluster-output-cdhit-id60-cov60.txt]
- ⇨ scripts/misc-scripts/run-recluster-ProteinOrtho.sh [This will produce: recluster-output-proteinortho-id60-cov60.txt]

6. Compare clusters from CD-HIT and ProteinOrtho
- ⇨ get-jaccard-index -i recluster-output-cdhit-id60-cov60.txt -j recluster-output-proteinortho-id60-cov60.txt

<u>Identification of Core Genome and Softcore Genome</u>

The Core Genome as well as the Softcore Genome can be obtained from the file "recluster-output-cdhit-id60-cov60.txt" (for CD-HIT) or "recluster-output-proteinortho-id60-cov60.txt" (for ProteinOrtho).
The first column indicates the number of genomes containing the given gene family (GF). Core genome genes are expected to be present on all genomes, therefore, all gene families with the number 1324 in the first column belong to the core genome. Accordingly, if the definition of softcore genome is 95% presence in all genomes, then the number of genomes in the first column should be at least 0.95 x 1324 genomes = 1257.8 genomes (i.e. 1258 genomes).

<u>ClermonTyping, Serotyping, Virulence Factor Typing, Resistance Typing</u>

ClermonTyping

- ⇨ mlst.py -i GCF_000005845.1.fasta -s ecoli -p mlst_db -o GCF_000005845.mlst -x -mp blastn

Serotyping

- ⇨ serotypefinder.py -i GCF_000005845.1.fasta -p serotypefinder_db -o GCF_000005845.serotype -x -mp blastn

Virulence Factor Typing

- ⇨ virulencefinder.py -i GCF_000005845.1.fasta -p virulencefinder_db -l 0.6 -t 0.8 -d "virulence_ecoli" -o GCF_000005845.vir -x -mp blastn

Resistance Typing

- ⇨ run_resfinder.py -ifa GCF_000005845.1.fasta -s "Escherichia coli" -l 0.6 -t 0.8 --acquired --point -b blastn -k kma -db_res_kma db_resfinder_kma -db_point_kma db_pointfinder -o GCF_000005845.resfinder

<u>Coincidence Analysis</u>
Run MUSCLE: Multiple Sequence Alignment of the seven housekeeping genes for all 674 genomes

- ⇨ muscle -in 7genes-674genomes.fna -out 7genes-674genomes.msa -maxiters 1 –diags

Run snp-sites: identifying SNPs in the MSA file

- ⇨ snp-sites 7genes-674genomes.msa

Run raxmlHPC: phylogenetic tree construction

- ⇨ raxmlHPC -f a -# 20 -s 7genes-674genomes.msa.snp_sites.aln -m GTRCAT -n tree -p 12345 -x 12345

CoinFinder: coincident association analysis

- ⇨ coinfinder -i gene-info-proteinortho-674genomes.txt -p RAxML_bestTree.tree -o coincident-po-associate -L 1e-20 -x 7 –associate
